# Biomass-specific rates as key performance indicators: A nitrogen balancing method for biofilm-based electrochemical conversion

**DOI:** 10.3389/fbioe.2023.1096086

**Published:** 2023-01-19

**Authors:** Marijn Winkelhorst, Oriol Cabau-Peinado, Adrie J.J. Straathof, Ludovic Jourdin

**Affiliations:** Department of Biotechnology, Faculty of Applied Sciences, Delft University of Technology, Delft, Netherlands

**Keywords:** biomass-specific rates, biofilm, electroactive bacteria, bioelectrochemistry, chain elongation, CO2 conversion, continuous bioreactors, microbial electrosynthesis

## Abstract

Microbial electrochemical technologies (METs) employ microorganisms utilizing solid-state electrodes as either electron sink or electron source, such as in microbial electrosynthesis (MES). METs reaction rate is traditionally normalized to the electrode dimensions or to the electrolyte volume, but should also be normalized to biomass amount present in the system at any given time. In biofilm-based systems, a major challenge is to determine the biomass amount in a non-destructive manner, especially in systems operated in continuous mode and using 3D electrodes. We developed a simple method using a nitrogen balance and optical density to determine the amount of microorganisms in biofilm and in suspension at any given time. For four MES reactors converting CO_2_ to carboxylates, >99% of the biomass was present as biofilm after 69 days of reactor operation. After a lag phase, the biomass-specific growth rate had increased to 0.12–0.16 days^−1^. After 100 days of operation, growth became insignificant. Biomass-specific production rates of carboxylates varied between 0.08–0.37 mol_C_ mol_X_^−1^d^−1^. Using biomass-specific rates, one can more effectively assess the performance of MES, identify its limitations, and compare it to other fermentation technologies.

## 1 Introduction

In recent years, Microbial Electrochemical Technologies (METs) gained substantial interest as innovative methods to replace fossil fuel based technologies and processes such as energy and chemicals production (Wang and Ren, 2013). METs exploit microorganisms by utilizing solid-state electrodes as either electron sink or electron source. To date, most studies on METs determine their performance by determining titers, current density or production rates normalized to volume (catholyte, cathode chamber or electrode volume) or electrode surface area (Patil et al., 2015; Fruehauf et al., 2020; Jourdin and Burdyny, 2021). While these performance indicators are important from an engineering perspective and to determine the technologies’ readiness level, they provide limited information on the actual metabolic activity. While the microorganisms perform the reaction(s) of interest, replicate, die, and wash-out, their amount changes over time. Traditional fermentation studies report performance and rates normalized to the amount of microbial biomass (X) in the reactor at any given time, i.e., biomass-specific rates of production or consumption (e.g., *q*_i_ in mol_i_ mol_x_^−1^ h^−1^ or g_i_ g_x_^−1^ h^−1^) (Heijnen et al., 1979; Rice, 1984; Wechselberger et al., 2013). This allows to assess the performance of the microbial catalyst under any condition. Similarly, chemo-catalytic electrochemical systems or other catalytic processes typically report the amount of catalyst used. The same approach should be followed for METs.

One MET of interest is microbial electrosynthesis (MES). In MES, microorganisms capable of reducing CO_2_ into valuable organic compounds such as carboxylic acids and alcohols are grown in a bioreactor in the presence of a cathode (Rabaey and Rozendal, 2010). This cathode supplies electrons for the CO_2_ reduction by the microorganisms. To date, the only experimental study reporting a biomass-specific growth rate in MES is from Sydow et al. (2017), who derived a biomass-specific growth rate *μ* = 2.16 days^−1^ for *Cupriavidus necator*. They measured the amount of planktonic biomass (i.e., microorganisms in suspension) by calibrating cell dry mass with optical density at 600 nm. However, this method is only applicable to systems using planktonic cells. Cabau-Peinado et al. (2021) constructed a generalized model for biofilm-driven MES of carboxylates from CO_2_ and derived *μ* = 0.12 days^−1^ based on the open culture system of Jourdin et al. (2019a). The model showed that the microbial rates were probably kinetically limited by CO_2_ availability even though dissolved CO_2_ was far from being depleted during the first 100 days. After 100 days, the system became limited by product toxicity, mainly from acetate and butyrate. These findings show that invaluable fundamental insights on the performance of the microorganisms can be derived from biomass-specific rates, also referred to as *q*-values. The real impact of variables such as operating conditions, electrode composition, and reactor design can be assessed from *q*-values. Consequently, there is a need for a low-cost *operando* method for quantifying biomass amount retained in the system to determine *q*-values at any given time in biofilm-based METs. *Operando* methods refer to methods used to describe systems over time in a non-destructive manner (Weckhuysen, 2002).

Several methods exist to quantify biomass amount, including in biofilm studies, such as dry weight measurements (Sydow et al., 2017), qPCR (Magalhães et al., 2019), optical density measurements (Sydow et al., 2017), protein content (Babanova et al., 2017), flow cytometry (Vignola et al., 2018), optical coherence tomography (OCT) (Molenaar et al., 2018; Hou et al., 2019), magnetic resonance imaging (Wolf et al., 2002; Häuser et al., 2022), cell counting using microscopy (Eddie et al., 2016; Phillips et al., 2020; Relucenti et al., 2021) or by cryo-sectioning thin biofilm slices (Huang et al., 1996; Franklin et al., 2015; Persson et al., 2017). However, these techniques suffer from key limitations to determine time-dependent *q*-values in biofilm-based systems. Most prominently, several of these techniques are destructive, allowing only one data point for biofilm biomass quantification at the end of operation. Tracking optical density of the fermentation broth allows non-destructive cell density determination over time, but only of microorganisms in suspension. OCT does allow tracking of the amount of biofilm over time, but only on 2D surfaces and the equipment is costly and requires a specific experimental design as well as specialized skills and expertise of the operator (Hackbarth et al., 2020).

Several of the aforementioned techniques to determine biomass amount are compromised in reactors fitted with 3D electrodes. Biofilm coverage might not be equally thick throughout the cathode due to regional differences in porosity (especially in fibrous 3D electrodes such as carbon felt), preferred flow patterns, and sheer stress. These can significantly alter the biofilm density and thickness, and become dynamic due to biofilm growth and its intrinsic effect on porosity (Stewart, 2012; Bottero et al., 2013). For example, Jourdin et al. (2018), who forced their catholyte to flow through a carbon felt cathode to overcome mass transfer limitations in their MES system, visually observed that a thick biofilm developed on the membrane-side of the electrode and a less thick biofilm on the outflow side of the electrode. Moreover, they described full biofilm coverage of the carbon felt fibers inside the electrode. To the best of our knowledge, biomass-specific rates have not been experimentally determined in biofilm-based METs.

The purpose of this study was to develop a simple method to experimentally derive biomass-specific rates in biofilm-based METs and to show its usefulness. A biofilm-based microbial electrosynthesis system (bMES) was used as case study here. The developed method consists of determining the amount of biomass present in the system, as biofilm and in suspension, at any given time, using total nitrogen and optical density (OD_600 nm_) measurements. To demonstrate the need for biomass-specific rates in bMES, biomass-specific production rate (*q*_*p*_) and biomass specific growth rate (*μ*) were experimentally determined and used to assess the microorganisms’ performance during bMES by comparing with other relevant technologies, i.e., syngas fermentation and chain elongation fermentation.

## 2 Materials and methods

### 2.1 Microbial electrosynthesis reactor operation

Four identical bioelectrochemical reactors were used (R1 to R4), each with a 7.35 cm^3^ piece of unmodified carbon felt (CGT Carbon, Germany) as cathode (7.35 cm^2^ projected surface area, 1 cm thickness as supplied by the manufacturer). The carbon felt volume of 7.35 cm^3^ was chosen to allow fast full biofilm colonization. An overview of the reactor and cathode dimensions can be found in the Supplementary Material I. Prior to use, carbon felt was cleaned by submerging it in 1 mol L^−1^ HCl and 1 mol L^−1^ NaOH for 24 h and subsequently treated with UV/ozone (Novascan, United States) for 45 min. A titanium wire (Advent Research Materials, United Kingdom) of 7 ± 0.5 cm was weaved through the carbon felt as current collector. To improve the conductivity between carbon felt and wire, a conductive coating was applied where the wire entered and exited the carbon felt, and was left to dry in an oscillator for 2 days. Each reactor was operated continuously for 194 days with a hydraulic retention time (HRT) of 8 days (*F*_in_ = 0.625 mL h^−1^) and a total catholyte volume of $V_{T}^{C}$ = 0.12 L. The medium was continuously circulated at a flow rate of 4.1 L h^−1^ (derived from Jourdin et al. (2019b)) The catholyte medium consisted of 0.4 g L^−1^ NH_4_Cl, 0.12 g L^−1^ MgCl_2_.6H_2_O, 0.06 g L^−1^ CaCl_2_.2H_2_O, 0.9 g L^−1^ Na_2_HPO_4_, 8.1 g L^−1^ KH_2_PO_4_, 4.5 g L^−1^ BrCH_2_CH_2_SO_3_Na and 2 mL L^−1^ trace nutrient medium. BrCH_2_CH_2_SO_3_Na was used as methane inhibitor. The trace nutrient medium consisted of: 10 g L^−1^ EDTA, 1.5 g L^−1^ FeCl_3_.6H_2_O, 0.15 g L^−1^ H_3_BO_4_, 0.03 g L^−1^ CuSO_4_.5H_2_O, 0.18 g L^−1^ KI, 0.12 g L^−1^ MnCl_2_.4H_2_O, 0.06 g L^−1^ Na_2_MoO_4_.2H_2_O, 0.12 g L^−1^ ZnSO_4_.7H_2_O, 0.15 g L^−1^ CoCl_2_.6H_2_O and 0.023 g L^−1^ NiCl_2_.6H_2_O. At day 62, the catholyte solutes, except the phosphates and methane inhibitor, were doubled in concentration to avoid possible nutrient limitations. Moreover, a gas mixture of CO_2_:N_2_ 50:50 was continuously bubbled at a rate of 100 mL min^−1^ through the catholyte in a bubble column. A titanium plate with a platinum-iridium coating (Ti Pt/Ir MMO, Magneto, Netherlands) was used as anode. The anolyte composition was similar to the catholyte composition, but excluded trace nutrients and methane inhibitor. Furthermore, the anolyte pH was corrected to pH ∼ 1.8 using 87% H_3_PO_4_ (approximately 10 mL per L anolyte) in order to favor protons crossing over the membrane over other cations. The cathode and anode compartments were separated by a cation exchange membrane (CEM, Membrane International, United States). pH was controlled at 5.80 ± 0.03 using either 1 mol L^−1^ NaOH or 1 mol L^−1^ HCl titration, with a pH probe (Prosense, Netherlands) attached to a PID system (JUMO, Germany). The reactors were operated inside a temperature controlled cabinet at 31 ± 1°C and kept in the dark to avoid potential phototrophic growth. At day 0 all reactors were inoculated with ± 460 mg L^−1^ biomass, obtained from cryogenic stocks of previously long-term operated MES reactors by Jourdin et al. (2019a). The inoculum was derived from biofilm as well as from planktonic cells. The electrochemical studies were controlled by a VMP3 Multichannel potentiostat (BioLogic, France) using an Ag/AgCl 3 mol L^−1^ KCl reference electrode (Prosense, Netherlands). During long-term operation, the cathodes were polarized in potentiostatic mode at −0.85 V vs. SHE (standard hydrogen electrode). Unless otherwise mentioned, all potentials are reported *versus* SHE in this manuscript.

### 2.2 Maintenance events

On day 42 and on day 52 of the experiment, electricity was switched off for 3 h (no gas feed, heat control, pH control, liquid recirculation or potential control by the potentiostat) due to maintenance (events I and II, repectively). To prevent acidification of the cathode chamber due to proton crossover, the anolyte was drained and refilled with the same composition, except for phosphoric acid, which was not added in order to maintain a pH of 5.8. After the power restart, the anolyte was changed again to its normal composition described earlier.

### 2.3 Analytical methods

A catholyte sample of 5 mL was taken twice a week from all reactors after inoculation. 100 µL was used to measure alcohols and carboxylic acids by GC-FID (Thermofisher, United States) with a Stabil-waxTM column of 25 m × 0.2 µm ID. The column was kept at 50°C for 7 min, ramped to 180°C in 8 min and kept at this temperature for 9 min. Helium was carrier gas at 1 mL min^−1^. Flame ionization detection was used at 250°C.

To investigate microbial growth, 2 mL catholyte was diluted ∼7.5x, filtered (0.2 µm), and the filtrate was analysed for total nitrogen using a TOC analyser coupled with a TN unit and auto sampler (TOC-L Series Total Organic Carbon Analyzer, Shimadzu, Japan). The oven temperature was set at 720°C. Optical density of the original undiluted sample was recorded at 600 nm (OD_600 nm_) to account for planktonic cells in the outflow of the system using a UV-VIS spectrophotometer (UV-1800 series, Shimadzu, Japan). The OD was calibrated to the nitrogen concentration in suspended biomass (planktonic cells, *c*_*N-pX*_ in mol L^−1^) in the catholyte. The calibration was obtained by the aforementioned total nitrogen analysis on a series of biomass (obtained from filtration of catholyte outflow on day 75) dilutions in catholyte without a nitrogen source. The derived calibration curve for concentration of nitrogen in planktonic biomass (mol L^−1^) was:

$$C_{N-pX}=0.0052\;*\;\left[{OD}_{600nm}\right]-0.00002$$ (1)

The obtained *R* ^2^ value for the calibration curve was 0.9989. The calibration data can be found in Supplementary Material (II).

### 2.4 Imaging

After terminating the reactors, three samples were taken from each biocathode using a sterile stainless steel knife under anaerobic conditions for viability analysis. For live/dead staining a FilmTracerTM LIVE/DEAD Biofilm Viability kit (InvitrogenTM) was used. The biofilm viability checker tool developed by Mountcastle et al. (2021) was used to quantify biofilm viability at the end of the experiments (Mountcastle et al., 2021). For imaging the stained samples a confocal laser scanning microscope system, LSM 710 (Zeiss Observer Z.1, Carl Zeiss), equipped with an AxioCam MRm camera was used. This LSM 710 system uses a Zeiss Observer Z.1 inverted microscope stand with transmitted light (HAL 100), UV (HBO 50), and laser illumination sources. The microscope is completely motorized with a motorized stage, z-drive (for focusing), objective turret. The samples were irradiated at excitation wavelengths at 488 nm and 543 nm for SYTO 9 and propidium iodine respectively, whereas the detection wavelengths were set to 493–578 nm and 566–797 nm respectively. The pinhole was set at 1 AU, and the detector gain at 500 and 700 for SYTO 9 and propidium iodine, respectively. For most images a Plan-Apochromat 20x/0.8 M27 objective was used, with the exception of the image for the R4 outflow sample where a Fluar 2.5x/0.12 M27 objective was used with a pinhole set at 0.68 AU.

### 2.5 Reactor performance determination

The mass balance for each reactor’s cathode compartment was defined as:

$$\frac{dn_{i}}{dt}=F_{in}c_{i,in}-F_{out}c_{i,out}+r_{i}V_{T}^{C}$$ (2)

Where *n*_*i*_ is the mole amount of compound *i*, *t* is time (d), *F* is the flow rate (L d^−1^), *c*_i,in_ is the ingoing concentration (0 mol_*i*_ L^−1^ for products in this study), *c*_i,out_ is the outgoing concentration, *r*_*i*_ is the volume-specific production rate of *i* (mol_i_ L^−1^ d^−1^) and $V_{T}^{C}$ is the total catholyte volume (L). The titrant flow, *F*_pH_ was much smaller than *F*_*in*_. Therefore, we disregarded it in this study, and we assumed *F*_in_ = *F*_out_ = *F*. Faradaic efficiency (FE%), or electron recovery, is defined as the total amount of electric charge retrieved in the products of interest (organics and biomass), *Q*_products_ (coulomb), divided by the total electric charge *Q*_T_ (coulomb) provided to the cathodic reaction:

$$FE\%=\frac{Q_{products}}{Q_{T}}\;*\;100\%$$ (3)

### 2.6 Biomass-specific rates determination

One method that does not suffer from being destructive and/or costly is using the elemental balances to quantify biomass amount and differentiate between planktonic and biofilm-based microorganisms. de Rink et al. (2022) used a nitrogen balance in their desulfurization process. They measured organic nitrogen using Hach kits to account for planktonic cells. Here, we measure total nitrogen to account for nitrogen assimilation into biomass, and calibrate optical density to nitrogen in planktonic cells to measure the planktonic cells amount at any given time as described in the analytical methods section. This prevents the needs for expensive testing kits for nitrogen species present in the medium. Biomass production was estimated based on a total nitrogen mass balance, assuming that nitrogen assimilation into biomass was the only relevant reaction involving elemental nitrogen. A schematic overview of the parameters used as well as a list of all parameters and subscripts used in the following equations can be found in supplementary material (II). Elemental nitrogen balances are used because the carbon balances include large terms for CO_2_ inflow and outflow, which will obscure carbon accumulation in biomass.

For total elemental nitrogen (N), balance Eq. 1 becomes:

$$\frac{dn_{N}}{dt}=Fc_{N-aq,in}-Fc_{N-aq,out}-Fc_{N-pX,out}$$ (4)

where *n*_*N*_ is the amount of total nitrogen in the cathode compartment, *c*_*N-aq,in*_ is the incoming dissolved nitrogen concentration (mol L^−1^), *c*_*N-aq,out*_ is the dissolved nitrogen concentration in the outflow (mol L^−1^), and *c*_*N-pXout*_ is the nitrogen content in planktonic cells in the outflow (mol L^−1^). No reaction rate *r* occurs in this equation as elemental nitrogen cannot be created nor destroyed. Integrating Eq. 4 for short time intervals Δ*t* between two sampling moments led to an equation for the amount of nitrogen accumulating in that interval:

$$∆n_{N}=\left(Fc_{N-aq,in}-Fc_{N-aq,out}-Fc_{N-pX,out}\right)\Delta t$$ (4a)

Overall nitrogen amount in the reactor over time was described by:

$$n_{N}=n_{N,0}+\sum ∆n_{N}$$ (5)

where $n_{N,0}$ is the initial mol amount of nitrogen, and ΣΔ*n*_*N*_ is the sum of the amounts of nitrogen accumulated between sampling moments. Assuming that the catholyte composition was similar to the measured outflow composition, the change in amount of dissolved N in the catholyte, obtained from multiplying *c*_*N-aq,out*_ by $V_{T}^{C}$ was negligible relative to the change in *n*_*N*_. Therefore, the change in nitrogen amount in the cathodic compartment was assumed to be due to uptake by biomass growth. Consequently, assuming *ν*_*N,X*_ = 0.2 mol_N_ mol_X_^−1^ as coefficient of nitrogen in the elemental formula of dry biomass, (Popovic, 2019) the total amount of biomass in the reactor was obtained from:

$$n_{X,T}=\frac{-n_{N}}{\nu_{N,X}}$$ (5a)

The concentration of planktonic cells biomass *c*_*pX*_ (mol L^−1^) in the catholyte due to planktonic cell growth and detachment of cells from the biofilm was obtained from:

$$c_{pX}=\frac{c_{N-pX}}{\nu_{N,X}}$$ (6)

Biomass-specific rates ( $q_{i}$ in mol_i_ mol_X_ d^−1^, including *μ*) were calculated using:

$$q_{i}=\frac{r_{i}V_{T}^{C}}{n_{X,T}}$$ (7)

Moreover, after multiplying *c*_*pX*_ by $V_{T}^{C}$ to obtain the amount *n*_*pX*_ of planktonic cells in the system, the amount *n*_*bX*_ of biofilm-based biomass in the system was obtained from:

$$n_{bX}={n_{X,T}-n}_{pX}$$ (8)

## 3 Results and discussion

Four reactors were operated under identical conditions as described in the materials and methods in order to derive q-values in biofilm-based microbial electrosynthesis. These reactors were used as benchmark reactors based on previous work (Jourdin et al., 2018; Jourdin et al., 2019a). For the purpose of benchmarking, the performance of these reactors are shown in Table 1 normalized to conventionally used key performance indicators in MES at pseudo steady states. As these pseudo steady states occurred at different times, the selected days vary among reactors. The time-dependent performance of all reactors are shown in Supplementary Material III. An extended version of Table 1 can be found in Supplementary Material IV.

**TABLE 1:** Conventional key performance indicators in MES: concentration, production rates and current densities. Selected time periods for R1: days 71–101, for R2: days 75–118, for R3 days 54–92, and for R4 days 141–198. Reactors were operated for 194 days with a HRT of 8 days. C_2_, C_4_ and C_6_ refer to acetate, butyrate, and hexanoate respectively. PSA: projected surface area.

		Concentrations	Production rates			Current densities	
		**g L** ^**−1**^	**Catholyte volume (g L** ^**−1**^ **d** ^**−1**^ **)**	**Electrode volume (g L**_**cathode**_^**−1**^ **d** ^**−1**^ **)**	**Projected surface area (g m** ^**−2**^_**PSA**_ **d** ^**−1**^ **)**	**Projected surface area (A m** ^**−2**^_**PSA**_ **)**	**Cathode volume (kA m** ^**−3**^ **)**
R1	C_2_	5.71 ± 0.32	0.74 ± 0.13	12.1 ± 2.1	121 ± 21	−75.17 ± 10.50	−7.52 ± 1.05
	C_4_	3.62 ± 0.36	0.48 ± 0.11	7.78 ± 1.85	77.7 ± 18.5		
	C_6_	0.08 ± 0.01	0.09 ± 0.02	1.52 ± 0.28	15.2 ± 2.8		
R2	C_2_	4.25 ± 0.32	0.54 ± 0.11	8.8 ± 1.9	88 ± 19	−22.19 ± 2.64	−2.22 ± 0.26
	C_4_	0.87 ± 0.21	0.11 ± 0.05	1.76 ± 0.78	17.6 ± 7.8		
	C_6_	0.09 ± 0.08	0.01 ± 0.03	0.18 ± 0.44	1.8 ± 4.4		
R3	C_2_	4.96 ± 0.16	0.64 ± 0.12	10.4 ± 2.0	104 ± 20	−25.55 ± 2.08	−2.55 ± 0.21
	C_4_	0.90 ± 0.18	0.11 ± 0.04	1.78 ± 0.67	17.8 ± 6.8		
	C_6_	n.a	n.a.	n.a.	n.a.		
R4	C_2_	9.37 ± 0.60	1.17 ± 0.17	19.1 ± 2.8	191 ± 28	−58.56 ± 13.39	−5.86 ± 1.34
	C_4_	7.99 ± 0.92	1.04 ± 0.24	17.0 ± 4.0	170 ± 40		
	C_6_	0.72 ± 0.11	0.010 ± 0.03	1.55 ± 0.43	15.5 ± 4.3		

### 3.1 Operational conditions and the inoculation of an enriched culture allowed hexanoate production after 30 days

Throughout the experiment acetate, butyrate and hexanoate were the only products measured in relevant amounts. Small peaks for propionate and valerate were observed irregularly, but always below the measurement limit. No alcohol peaks were observed. The first acetate production was recorded in R1 and R3 after 13 days, immediately reaching 1.08 g L^−1^ and 0.46 g L^−1^ respectively. In this study a 50:50 CO_2_:N_2_ ratio was used *versus* 30:70 in previous work. R2 started producing organics on day 19. However, R4 only started producing organics on day 82. The reason behind this observed lag phase is not fully clear, but could be explained by contamination of the reactor by competing microorganisms, as biomass growth as well as current consumption were still observed during the first 82 days. On the same day as acetate was first measured, 230 mg L^−1^ and 210 mg L^−1^ butyrate was recorded in R1 and R3 respectively. This was surprising as according to previous work the threshold C_2_ concentration triggering butyrate production was 2.5–4 g L^−1^ (Jourdin et al., 2018). The start of hexanoate production did match with the threshold of 0.5–2.5 gC_4_ L^−1^ observed in said study. It was first measured after 29 days in R1, 77 days in R2, and 131 days in R4. In R3 hexanoate was only recorded in 2 data points (day 103 and day 106). When comparing the data from Table 1 with literature, it can be deduced that all reactors performed in accordance with commonly derived numbers for biofilm-based MES using 3D cathodes (Flexer and Jourdin, 2020; Prévoteau et al., 2020). The methane inhibitor BrCH2CH2SO3Na (2-BES) was used in this study, which was postulated to function as electron acceptor to oxidize ethanol to CO_2_ by *Azospira Oryzae* by Steinbusch et al. (2011). Future research should address if 2-BES affects performance in biofilm-based MES systems. The results show that the reactors were able to produce relevant concentrations of carboxylates up to hexanoate, making them suitable as benchmark systems to determine biomass-specific rates for biofilm-based MES reactors.

### 3.2 The amount of biomass retained in the reactors deviated by a factor of 2 after full colonization of the cathode was achieved

**FIGURE 1:**
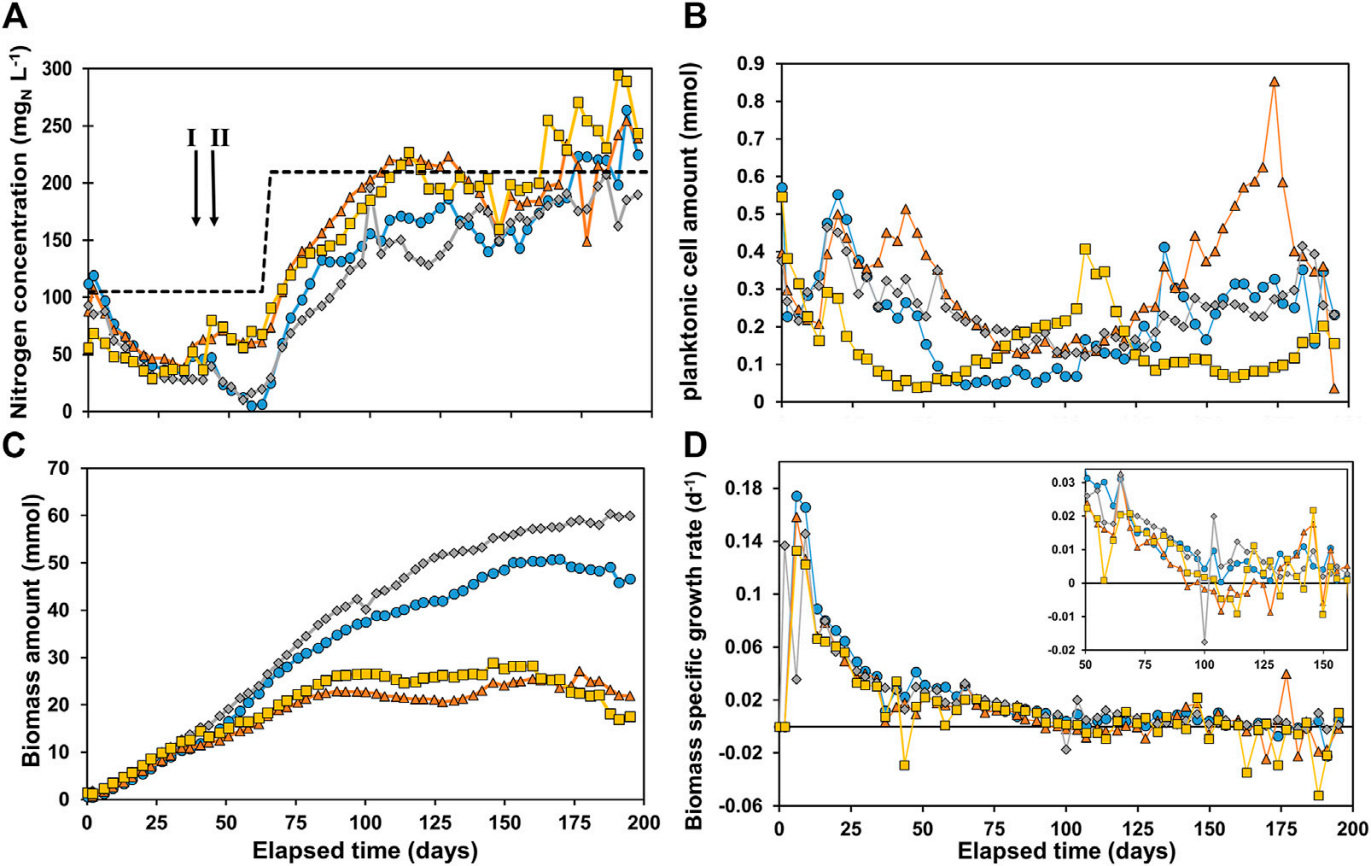
**(A)** Nitrogen concentration in the reactors, with the total nitrogen inflow concentration as dashed line **(B)** planktonic cell amount in the reactor, **(C)** total biomass amount in the reactor and **(D)** specific growth rate *μ*. R1 is in blue circles, R2 in orange triangles, R3 in grey diamonds, and R4 in yellow squares. The arrows in Figure 1A indicate event I and II as discussed in the materials and methods section.

In Figure 1, the total nitrogen concentration (A), calculated planktonic cell amount (PCA) (B), calculated total biomass amount (C) and derived µ-values (D) are shown for all four reactors.

**FIGURE 2:**
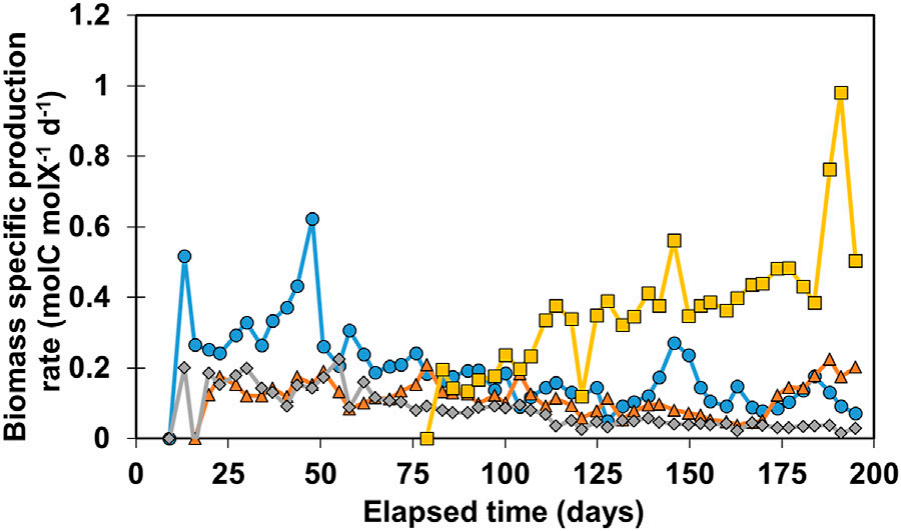
Calculated *q*_*p*_ values for all reactors. R1 is in blue circles, R2 in orange triangles, R3 in grey diamonds, and R4 in yellow squares.

A decrease in nitrogen concentration was measured in all reactors, indicating nitrogen consumption (Figure 1A). On day 62 the soluble total nitrogen in R1 and R3 was almost depleted. Hence, it was decided to double the nutrient concentration to prevent limitation. This was implemented in all reactors to maintain similar conditions. After ∼100 days the ammonium concentration was close to its feed concentration, indicating limited growth. After 160 days the nitrogen concentration measured was higher than the influent NH_4_^+^-N concentration in R4, with R2 and R1 following on day 167 and 170 respectively. The reason for this was most likely cell lysis of non-viable cells and/or extracellular polymeric substances (EPS) releasing soluble nitrogen compounds (proteins, amino acids and NH_4_^+^). Figure 1B shows an initially decreasing concentration of planktonic cell amount (PCA) in the reactor. This can be caused by: 1) washout *via* the effluent and/or 2) biomass attachment to the electrode and consequently biofilm formation. An increasing PCA can be caused by 1) more planktonic cells growth and/or 2) cell detachment from the biofilm. The effect of these phenomena cause significant different trends in PCA in all reactors. After 110 days the PCA started to gradually increase in R2 and performance was deteriorating (see Supplementary Material IIIb). After 173 days the reactor PCA sharply decreased and performance spiked. The reason for this observation is not clear, but it was suspected that something in the system caused substantial resistance which was suddenly reduced as cathodic current significantly increased on day 173. The amount of biomass in all reactors (Figure 1C) was very similar until reaching 10.9 ± 1.2 mmol_X_ around day 30. From this point onwards, the amount of biomass started to deviate between R1 + R3 and R2 + R4. R1 and R3 reached significantly higher values, plateauing at 50.0 ± 1.0 mmol_X_ and 59.2 ± 0.8 mmol_X_, respectively, both after ∼150 days. R2 and R4 plateaued much earlier and at lower values, after about 85 days at 23.1 ± 1.6 and 26.5 ± 1.1 mmol_X_, respectively. However, as these trends are not observed in the PCA in Figure 1B, the plateauing can be attributed to full colonization of the cathode by biofilm. The biomass specific growth rates (*μ*-values) are shown in Figure 1D. A lag phase of 3 days was observed in all reactors, after which the μ-value increased to 0.12–0.17 days^−1^. The μ-value steadily decreased for all reactors to 0.026 ± 0.004 days^−1^ after 50 days, and below 0.01 days^−1^ after 100 days. Yet, even after 100 days the difference in biomass amount in the reactors was increasing. This was caused by: 1) the *μ*-value being routinely higher in R1 and R3 than R2 and R4 over long periods as can be observed in the zoom in window in Figure 1D, and 2) The μ-value reaching negative values in R2 and R4 especially after 160 days as biofilm-based biomass decays and/or detaches. The results show that the reactors did not behave as replicates even though they were controlled at the same conditions. Due to the complexity of the systems and use of mixed culture the reactor performances are likely extremely sensitive to slight variations in operational conditions (e.g., exact applied potential, temperature, retention time, pH control, electrode packing and placement). The difference in performances between the four reactors deserves further investigation.

### 3.3 Biofilm accounted for >99% of biomass present in the reactors

The most plausible hypothesis for the total amount of biomass in the reactors reaching a plateau as shown in Figure 1C is biofilm saturation, in which space restriction prevents more biofilm growth. Subtracting the planktonic cells amount (Figure 1B) from the total amount of biomass (Figure 1C) gives the biomass retained in the system as biofilm at any time. In Supplementary Material V the ratio between biomass as planktonic cells and biofilm is shown, which shows that after 69 days > 99% of the biomass is in biofilm in all reactors. Photos of the cathodes (membrane side and outflow side) can be found in the Supplementary Material VI. These images show full coverage of the carbon felt, but the thickness of the biofilm varies. The variation in biomass amount per reactor may be due to the heterogeneity of the structural composition of the carbon felt. Moreover, the way the carbon felt electrode is placed and packed inside its chamber can unintentionally vary, affecting its porosity and thus the space available for biofilm formation. Consequently, the liquid flow through the carbon felt is also affected by its packing and placement, also impacting local mass transport and biofilm formation. The values of the concentrations of biomass when normalizing to electrode volume at the end of the experiments are 6.7, 3.7, 8.1, and 3.9 mmol_X_ cm^−3^_cathode_ for R1, R2, R3, and R4 respectively. These values are in a similar order of magnitude as predicted by Cabau-Peinado et al. (2021), who described microbial kinetics and reactor performance of a comparable MES system by computational modelling. In their model, the biomass concentration in the reactor plateaus after approximately 150 days, with a biomass concentration of 8.2 mmol cm^−3^_cathode_. In comparison, the theoretical biomass concentration is approximately 13 mmol cm^−3^ (based on a cell density of 1.09 g cm^−3^ and dry weight ratio of 30%) (Bakken and Olsen, 1983). This would mean that 29–63% of the physical space is occupied by biomass in the cathodes.

### 3.4 The biofilm colonization may be improved by growth medium engineering and enhancement of mass transport

As also found in this experimental study, μ-values reaching below 0.01 days^−1^ after 100 days of reactor operation resulted from model calculations by (Cabau-Peinado et al., 2021). This indicates that all reactors used in the present study reached a mature biofilm and organics production was most likely maintenance dictated. The derived biomass growth rates in this study are relatively low compared to growth rates found in related anaerobic fermentation technologies such as syngas fermentation and chain elongation. As a consequence, reaching a mature biofilm was relatively time consuming and requires improvement from an application point of view. Reported μ-values can widely vary due to suboptimal conditions for biomass growth. For acetogens grown on H_2_/CO_2_ μ-values are generally in the region of 1.2–2.9 days^−1^ (Heijnen et al., 1979; Groher and Weuster-Botz, 2016; Bengelsdorf et al., 2018; Acharya et al., 2019; Philips, 2020). The highest reported growth rate is by Groher and Weuster-Botz (2016), who reported a maximum biomass-specific growth rate of 5.77 days^−1^ for the acetogen *Terrisporobacter mayombei* grown on a H_2_:CO_2_ mixture using their developed growth medium specific to acetogens. Candry et al., obtained a maximum specific growth rate for *Clostridium kluyveri* of 2.9 days^−1^, a model organism frequently studied in carboxylate chain elongation using soluble electron donors (Candry et al., 2018). Allaart et al. found an average growth rate of 1.39 days^−1^ for an open culture when studying the effect of product inhibition in chain elongation using sequencing batch bioreactors (Allaart et al., 2021). In open cultures, generally lower growth rates are found, ranging between 0.12 days^−1^ to 2.9 days^−1^ (Roghair et al., 2016; Candry et al., 2020; Shrestha et al., 2022). The relatively low growth rates observed in our study may be attributed to absence of vitamins and/or yeast extract supplementation. Species dependent on these supplements must rely on interspecies supplementation of minerals and vitamins, potentially limiting their growth. Growth medium engineering could be performed in follow-up work in order to improve the start-up time and full colonization of the electrode (Ammam et al., 2016; Groher and Weuster-Botz, 2016). Moreover, the electron transfer mechanisms and mass transport of protons, hydroxide ions, nutrients, substrates, and products in cathodic biofilms should be studied more extensively as these may be contributing factors to growth limitations (Jourdin and Burdyny, 2021).

### 3.5 Biofilms are key in this system, but more does not necessarily result in higher volume-specific productivity

To study the performance of the reactor’s biofilms more extensively, biomass-specific production rates (*q*_*p*_) were determined over time on basis of moles of carbon in the three organic products jointly. The results are shown in Figure 2.

The general trend for R1-R3 shows a gradual decrease of *q*_*p*_ over time as the biofilm matures. Biomass-specific production rates in R1 increased until day 50, reaching 0.62 mol_C_ mol_X_^−1^d^−1^, and decreased rapidly to 0.24 mol_C_ mol_X_^−1^d^−1^. After 55 days the performance of R1 and R3 gradually decreased from 0.2 mol_C_ mol_X_^−1^d^−1^ to 0.12 mol_C_ mol_X_^−1^d^−1^ and 0.06 mol_C_ mol_X_^−1^d^−1^ respectively on day 128. R2 stayed relatively stable around 0.17 mol_C_ mol_X_^−1^d^−1^, until day 100, after which *q*_*p*_ decreased to 0.06 mol_C_ mol_X_^−1^d^−1^ on day 160. As production rates increased again, R2 reached 0.2 mol_C_ mol_X_^−1^d^−1^ at the end of the experiment. Remarkably, organics production was only recorded after 80 days of operation in R4. Contrary to the declining trend of *q*_*p*_ observed in the other reactors, q_p_ in R4 increased over time from 0.2 to 0.48 mol_C_ mol_X_^−1^d^−1^ on day 175. This increase of *q*_*p*_ can be explained by both an increase in volumetric performance during this time as well as a slight decrease in biomass amount retained in the system. It is also possible that this microbial community shifted towards enrichment of acetogens and chain elongators after 80 days. The highest *q*_*p*_ value was recorded in R4 on day 196 at 0.96 mol_C_ mol_X_^−1^d^−1^, after which it decreased again to 0.49 mol_C_ mol_X_^−1^d^−1^. No clogging of influent or effluent tubing was observed between day 183–195 which could have explained the two outliers. The results of Figure 2 illustrate that *q*_*p*_ is a variable that can fluctuate over time, showing a decreasing trend when biomass amount increased while production rates at reactor scale are relatively stable. More biomass present in the system does not necessarily mean higher volumetric productivity, especially in systems with active cell retention. For example, R4 retained three times less biomass than R3 (Figure 1C), however the biomass in R4 is 11.6 ± 3.2 times more active in terms of biomass-specific production rate than the biomass in R3 between day 150 and 183 (Figure 2).

### 3.6 Biomass-specific production rates in MES can be enhanced

In table 2 the average *q*_*p*_ of the MES reactors are compared to several syngas fermentation and chain elongation studies in order to assess whether the derived values for *q*_*p*_ are relevant or insignificant quantities. These studies were selected based on whether *q*_*p*_ was reported and/or sufficient data was provided to calculate it. Moreover, the studies are compared based on whether they utilize open or single cultures, on the substrate (s) used, and whether a biofilm was formed or only suspended cells were considered.

**TABLE 2:** Comparison of reactor performance with Syngas fermentation and Chain elongation fermentation. For studies normalizing biomass to dry cell weight (DCW), a molecular weight of 25.25 g mol^−1^ was used for biomass. For studies normalizing biomass to volatile suspended solids (VSS) it was assumed that 1 gVSS equals 1 gDCW. C_5_, C_7_ and C_8_ refer to pentanoate, heptanoate and octanoate respectively.

Technique	Culture	Biofilm/planktonic	Input composition	Main products	*q*_*p*_ (MolC molx^−1^d^−1^)	References
Microbial electrosynthesis	open	Biofilm	e^−^/CO_2_	Carboxylates C_2_,C_4_,C_6_	0.08–0.37	Average between reactors in this study
Gas fermentation	open	Biofilm	H_2_/CO_2_	Carboxylates C_2_,C_4_,C_6_,C_8_	0.31	Zhang et al. (2013)
Gas fermentation	pure	Planktonic	H_2_/CO_2_	acetate, ethanol	0.23	Ammam et al. (2016)
Syngas fermentation	pure	Planktonic	H_2_/CO/CO_2_	acetate, ethanol	0.81	Shen et al. (2014)
Syngas fermentation	pure	Planktonic	H_2_/CO/CO_2_	acetate, ethanol	9.49	Lee et al. (2019)
Syngas fermentation	pure	Planktonic	H_2_/CO/CO_2_	acetate, ethanol	2.94	Shen et al. (2020)
Syngas fermentation	pure	Planktonic	H_2_/CO/CO_2_	acetate, ethanol	7.58	Valgepea et al. (2017)
Syngas fermentation	pure	Planktonic	H_2_/CO/CO_2_	acetate, ethanol	12.4	Heffernan et al. (2020)
Syngas fermentation	pure	Planktonic	H_2_/CO/CO_2_	acetate, ethanol	0.73	Ahmed et al. (2006)
Syngas fermentation	pure	planktonic	H_2_/CO/CO_2_	acetate, ethanol	0.12	Phillips et al. (1993)
Chain elongation	pure	planktonic	acetate + ethanol	Carboxylates C_4_,C_6_,C_8_	0.26	Steinbusch et al. (2011)
Chain elongation	open	biofilm + planktonic	Acetate + ethanol	Carboxylates C_4_,C_5_,C_6_,C_7_	10.0	Roghair et al. (2016)

The table illustrates that in general, the performance of the MES reactors normalized to biomass amount was relatively low. The study most closely related to the current study is by Zhang et al. (2013), as they formed an open culture biofilm in a hollow fibre membrane bioreactor producing medium chained carboxylates up to caprylate (C8) from a CO_2_/H_2_ mixture. Their *q*_*p*_ is lower than the average *q*_*p*_ measured in R4, but higher than the average found in the other reactors. The highest *q*_*p*_ values were found in more recent single culture syngas fermentation studies, with the exception of the chain elongation study Roghair et al. (2016) In their study, they managed to form chain elongating granular sludge, allowing cell retention and applying relatively short hydraulic retention time, increasing steady-state soluble substrate concentrations, decreasing product inhibition, and therefore increasing production rates. This comparative analysis highlights that there is room to significantly improve metabolic rates in MES.

### 3.7 Limitations of this study

**FIGURE 3:**
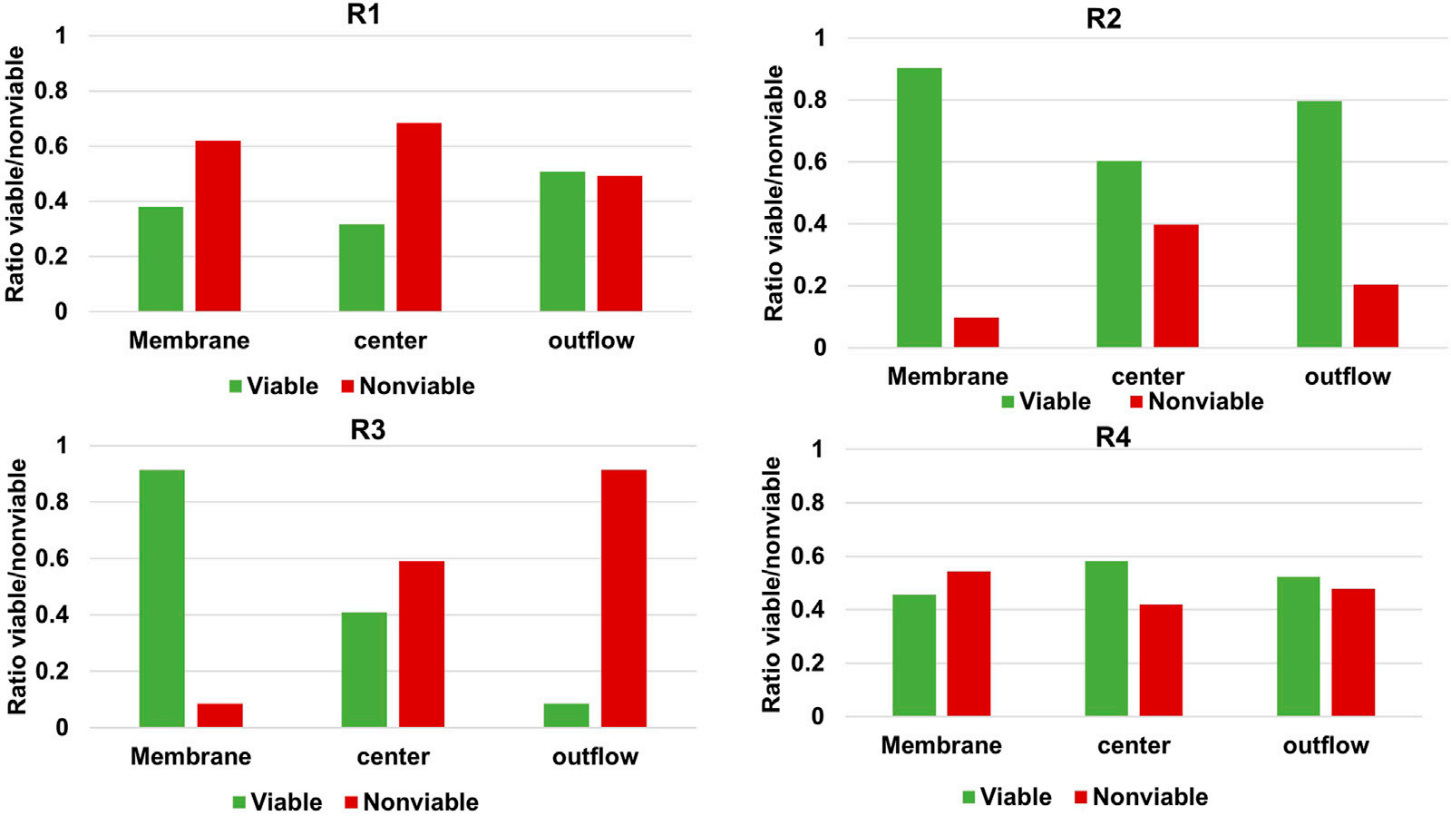
Relative abundance of live and dead microorganisms in the cathodic biofilm of each MES reactor at three different locations of the carbon felt.

Even though the method used in this study circumvents several disadvantages of more commonly used techniques, there are still some limitations. Most evident, the method relies on the assumption that the gap in the nitrogen balance when accounting for outflow of medium and suspended cells can be assigned to biofilm-based biomass. Several phenomena can potentially complicate this method. The identified complications relevant for the current study are nitrogen accumulation in extracellular polymer substances, nitrogen-containing salt precipitation and retained non-viable cells accumulating in biofilm. To determine if accumulation of non-viable cells in the biofilm may have caused an underestimation of *q*_*p*_, live/dead staining was performed at three different locations of the cathode for every reactor. Confocal images can be found in the Supplementary Material (VII) and were analysed for viable:non-viable ratio using Biofilm viability checker tool developed by Mountcastle et al. (2021) for ImageJ (Legland et al., 2016). The results of the analysis are illustrated in Figure 3.

Figure 3 shows that in all reactors a significant percentage of the biofilm stained as non-viable, but the extent varies greatly per reactor and per location. Izadi et al. (2020) found >90% of viable cells in their biofilm-based MES reactor when applying −1 V vs. Ag/AgCl and feeding CO_2_, after 104 days while refreshing 40% of the medium every 14–21 days. The higher non-viable cell ratio in our study may be explained by the longer operation of 194 days and diminished biomass growth rates as illustrated in Figure 1D. Based on the significant ratio of non-viable cells shown in Figure 3 it should therefore be noted that the biomass-specific rates (*q*_*p*_ and μ-values) of the reactors were indeed underestimated due to retained non-viable biomass in the biofilm. The main advantages of the method are that it allows *operando* monitoring of biomass amount present in the system in a non-destructive manner. Techniques to improve the accuracy, e.g., EPS and live/dead determination, can be considered as complementary. When applying this method to other METs, other limitations may be important as well. In this study, reducing conditions were used, but if ammonium can get oxidized to NO_x_ and/or elemental nitrogen, off-gas analysis may be required to close the nitrogen balance. In other cases low biomass quantity or low nitrogen content may compromise using the nitrogen balance. Moreover, in this study OD_600nm_ was used to calibrate nitrogen content in suspended cells, which is known to fluctuate over time even in pure cultures (Candry et al., 2018). This can be circumvented by updating the calibration over time. The impact of any change in the slope of the calibration curve presented for the systems used in this study is very low. This is due to the low optical density recorded, and thus low planktonic cell concentration, in comparison to total biomass amount retained in the reactors. However, other continuous systems with a higher ratio of planktonic biomass could be impacted to a larger extent as a higher or lower ratio of the biomass retained in the system would wash out. Moreover, in systems with much higher suspended cell densities *versus* biofilm cells or larger reactor volumes, other methods such as routine dry weight measurements of reactor broth may become viable methods as well.

### 3.8 Biomass specific rates are the true microbial performance indicator

When normalizing the production rate to projected surface area of the electrode (PSA), an average production rate of 214 ± 43 g m^−2^_PSA_ d^−1^ and current density of −75 ± 10 A m^−2^_PSA_ in R1 were found, which is within the top 5% of MES studies, as reported in the review by Flexer and Jourdin (2020). Even though current densities, production rates and titers reported in this study are reasonable compared to previous studies in MES, biomass specific production rates show that the microbial community is most likely facing limitations and is not performing to its full potential. This information is key in order to assess the true impact of changes such as in operational conditions, reactor configuration or electrode modifications on the microbial performance. Studying the biofilm as described in this study allows differentiating improvements in performance thanks to increased biomass quantity or to higher metabolic activity. Moreover, it allows comparing performance to other biotechnological processes such as syngas fermentation or chain elongation. Further research should focus on investigating what is limiting biofilm-based MES. As demonstrated in the results section, significant differences in performance were observed for all reactors. The reason for these differences are currently unknown and should be studied more extensively. Proposed research areas are cathode design (especially porosity) and interactions within the microbial community, using biomass specific rates as key performance indicators (growth, uptake and production rates).

## Data Availability

The original contributions presented in the study are included in the article/Supplementary Material, further inquiries can be directed to the corresponding author.
