# Supplementary material for: Biomass-specific rates as key performance indicators: A nitrogen balancing method for biofilm-based electrochemical conversion
Source: Front Bioeng Biotechnol. 2023 Jan 19;11:1096086. doi: 10.3389/fbioe.2023.1096086 (PMC9892193; doi:10.3389/fbioe.2023.1096086)
Supplement: Supplementary file 1 [file DataSheet1.docx]

**Supplementary Material for Biomass-specific rates as key performance indicators of microbial electrochemical technologies: a nitrogen balancing method for biofilm-based electrochemical conversion.**

Marijn Winkelhorst, Oriol Cabau Peinado, Adrie Straathof, and Ludovic Jourdin

# Schematic overview reactor set up


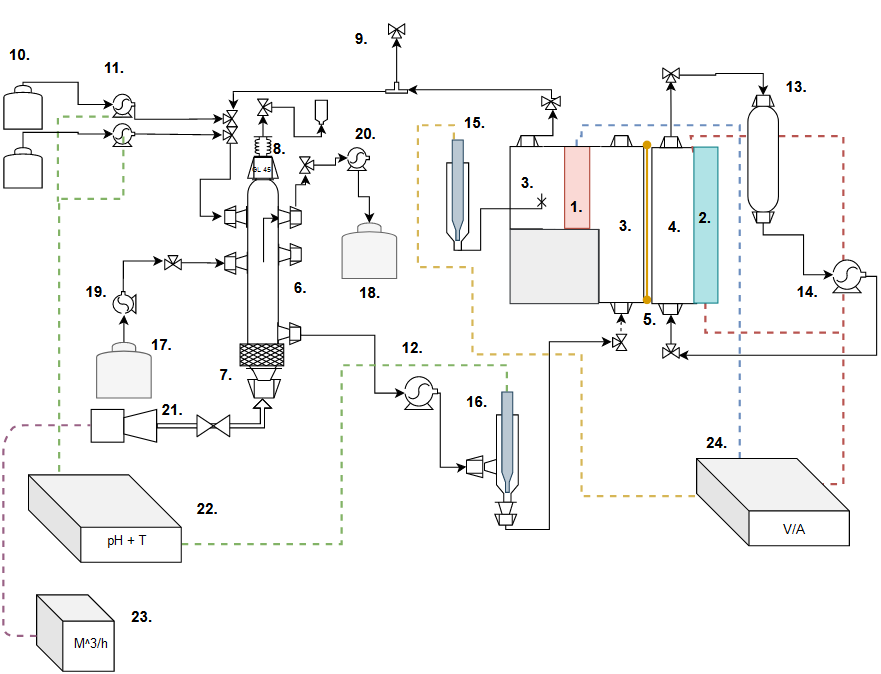


Figure S 1 Schematic overview of the reactor set up. The numbers represent: 1. Cathode, 2. Anode, 3. Cathode chamber, 4. Anode chamber, 5. (Cation exchange) membrane 6. Cathode medium recirculation column 7. Gas sparger, 8. Condenser, 9. Sampling point, 10. pH control flasks (1M HCl and 1M NaOH), 11. pH control pumps, 12. Cathode recirculation pump 13. Anode medium recirculation bottle, 14. Anode recirculation pump, 15. Reference electrode (Ag/AgCl 3M KCl), 16. pH meter+flask, 17. Influent bottle, 18. Effluent bottle, 19. Influent pump, 20. Effluent pump, 21. Mass flow controller, 22. pH and temperature controller, 23. Mass flow controller operator, 24. Potentiostat. The black arrows represent tubing, the black dotted arrow represents the potential “safety” tubing to bypass the cathode, the yellow dotted, blue dotted and red dotted lines represent the reference, cathodic and anodic cables respectively, the green dotted line represents the pH control cables and the purple dotted line represents the mass flow control cable.


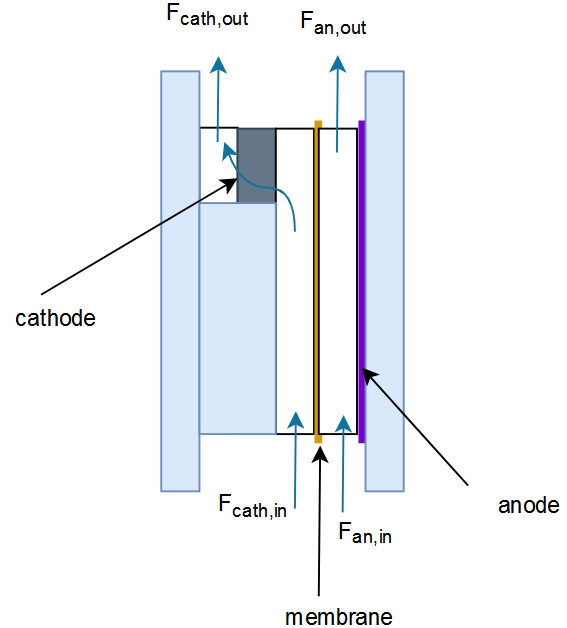


Figure S 2: schematic overview reactor cell with catholyte flow path


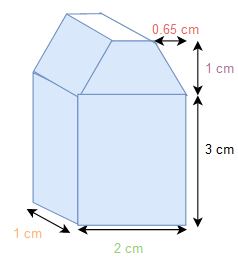


Figure S 3: Schematic overview of the cathode with its dimensions. The projected surface area equals 7.35 cm^2^, the volume equals 7.35 cm^3^

# Nitrogen balance

## Schematic overview parameters


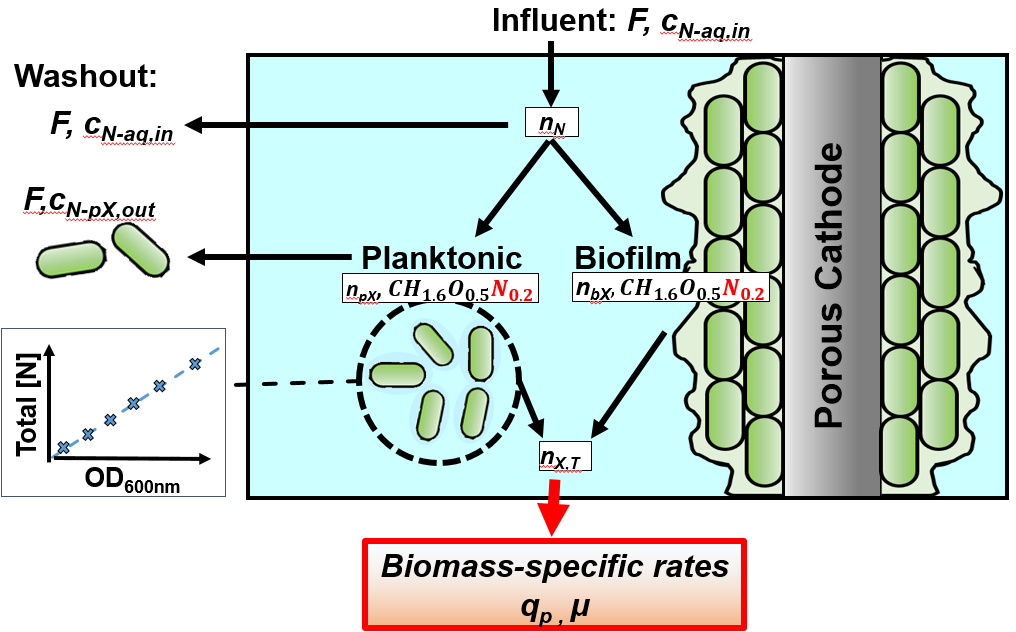


Figure S 4. Schematic overview parameters

## Parameter list & abbreviations

Table S 1 list of parameters

| Symbol | description parameter | Unit |
| --- | --- | --- |
| *n_i_* | mole amount of compound *i* | Mol*_i_* |
| *t* | time | d |
| *F* | Flow rate | L d^-1^ |
| *V_T_^c^* | Total catholyte volume | L |
| *r_i_* | Reaction rate of compound i | Mol_i_ d^-1^ |
| *c_i,in_* | Inflow concentration compound i | Mol_i_ L^-1^ |
| *c_i,out_* | outflow concentration compound i | Mol_i_ L^-1^ |
| *FE%* | Faradaic efficiency | % |
| *Q_products_* | amount of electric charge retrieved in the products of interest | coulomb |
| *Q_T_* | Total electric charge provided | Coulomb |
| *ν_i,y_* | Stoichiometric coefficient of i in y | dimensionless |
| *q_i_* | Biomass-specific production/consumption rate of compound i | mol_i_ mol_X_ d^-1^ |

Table S 2. List of subscripts used to discriminate parameters

| Subscript | Description subscript |
| --- | --- |
| *N* | Nitrogen |
| *N-aq,in* | Soluble nitrogen in inflow |
| *N-aq,out* | Soluble nitrogen in outflow |
| *N-pX,out* | Nitrogen content of planktonic biomass in outflow |
| *N,0* | Starting nitrogen content |
| *N,X* | Nitrogen content in biomass |
| *X,T* | Total biomass |
| *bX* | Biofilm biomass |
| *pX* | Planktonic biomass |

1. Nitrogen versus optical density calibration curve


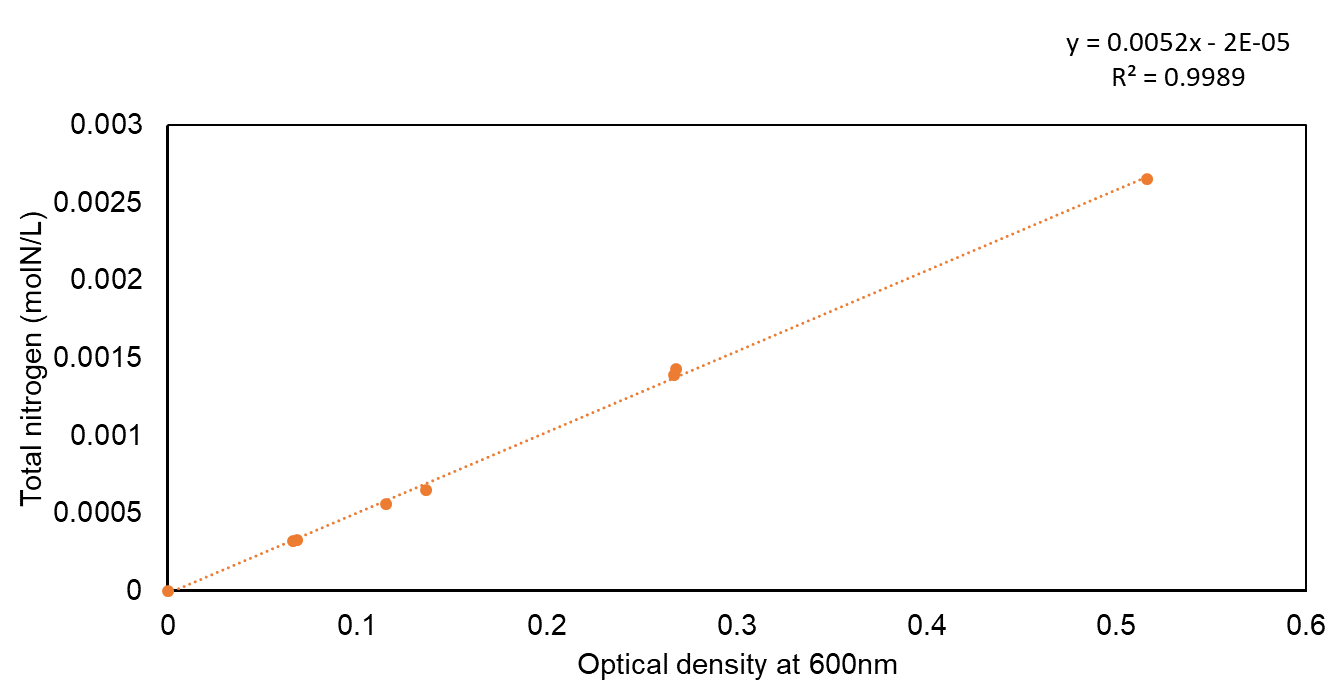


Figure S 5: calibration total nitrogen with optical density

# Reactor performance

## Measured optical density for all reactors


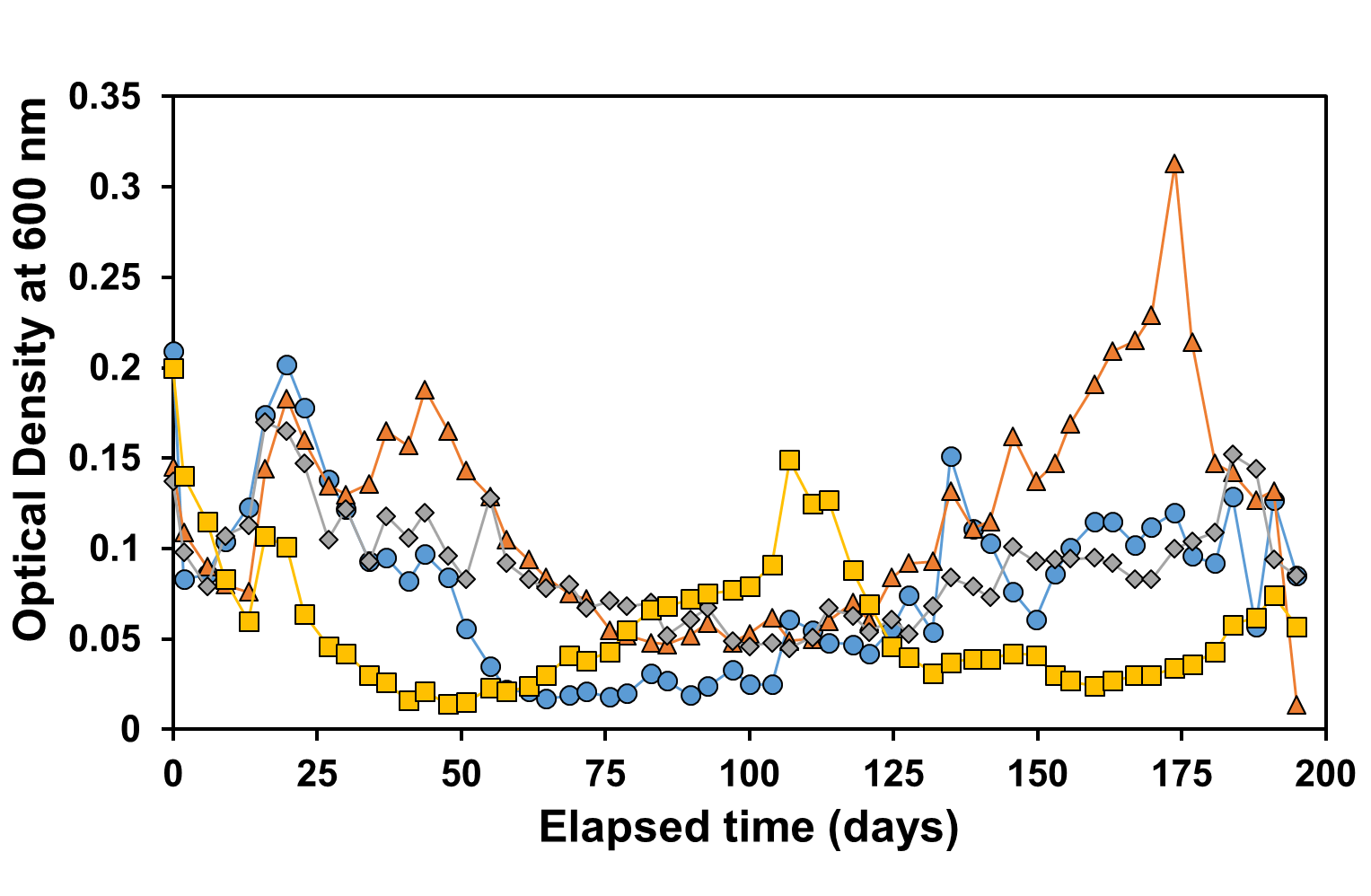


Figure S 6. Measured optical density at 600 nm for all reactors. R1 is in blue circles, R2 in orange triangles, R3 in grey diamonds, and R4 in yellow squares.

## Performance of R1


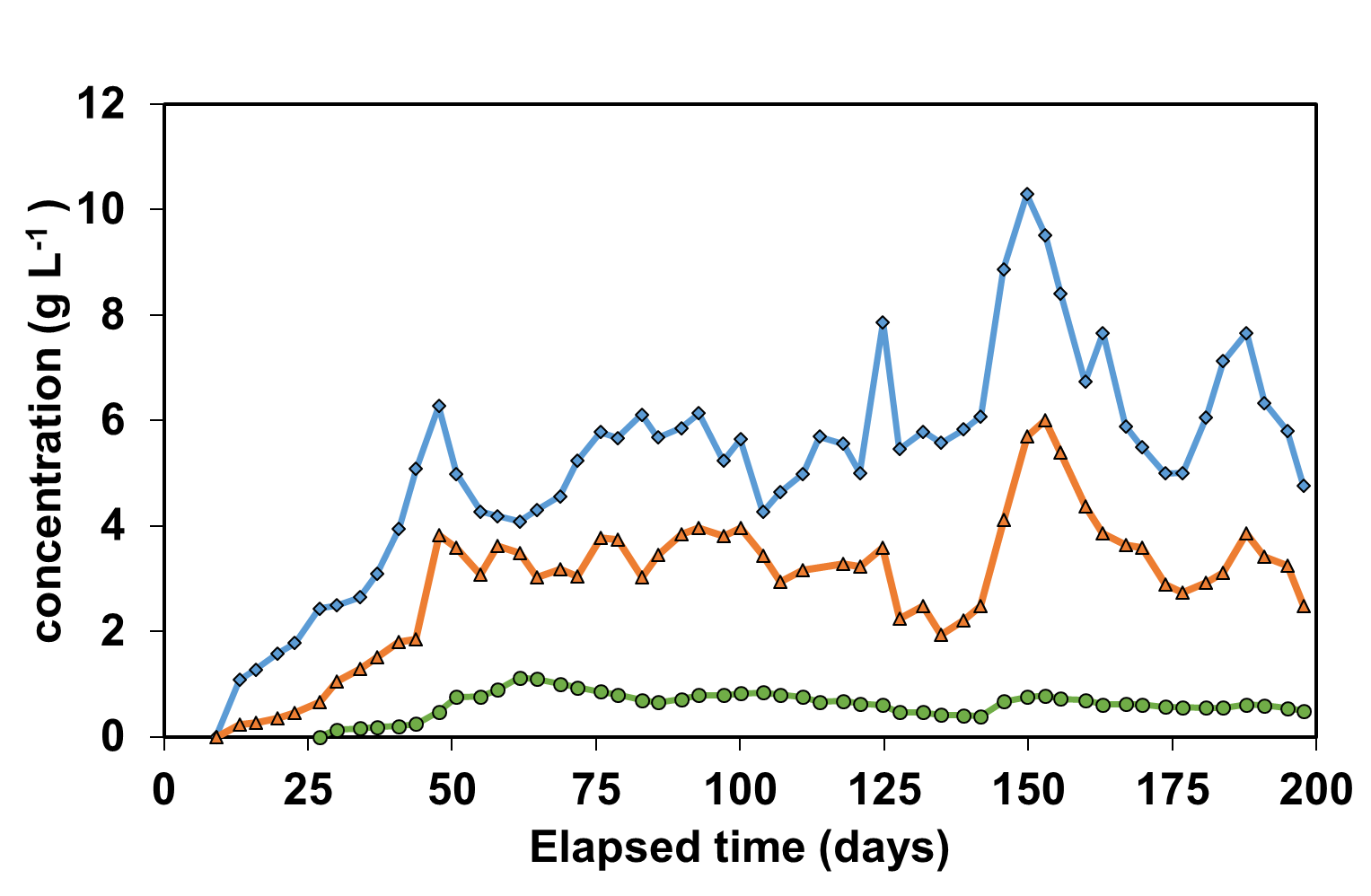


Figure S 7. Concentration of organics R1. Colour code: blue diamonds is acetate, orange squares is butyrate, and green circles is hexanoate


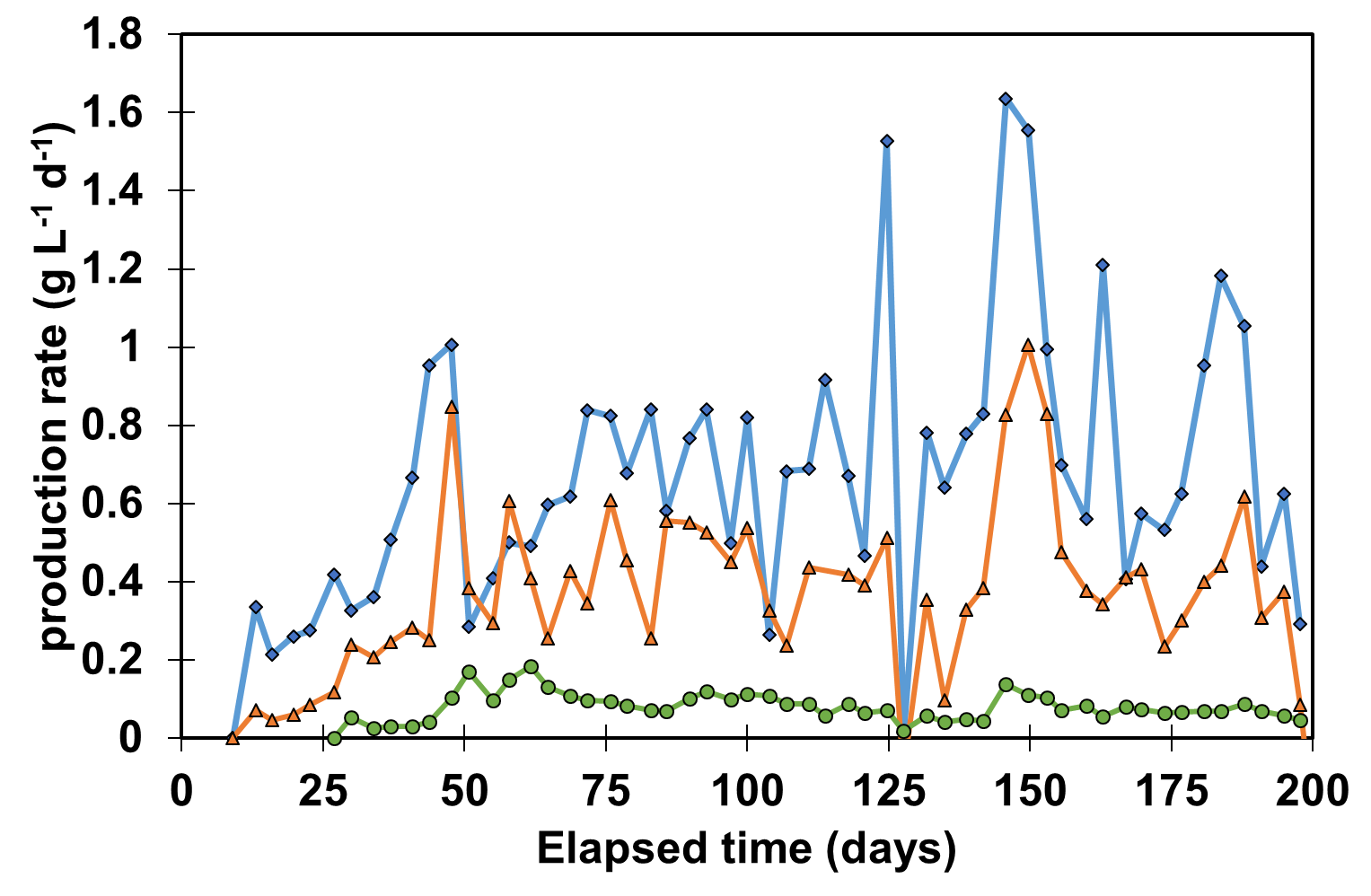


Figure S 8. Volume-specific production rate normalized to total catholyte volume in R1. Colour code: blue diamonds is acetate, orange squares is butyrate, and green circles is hexanoate


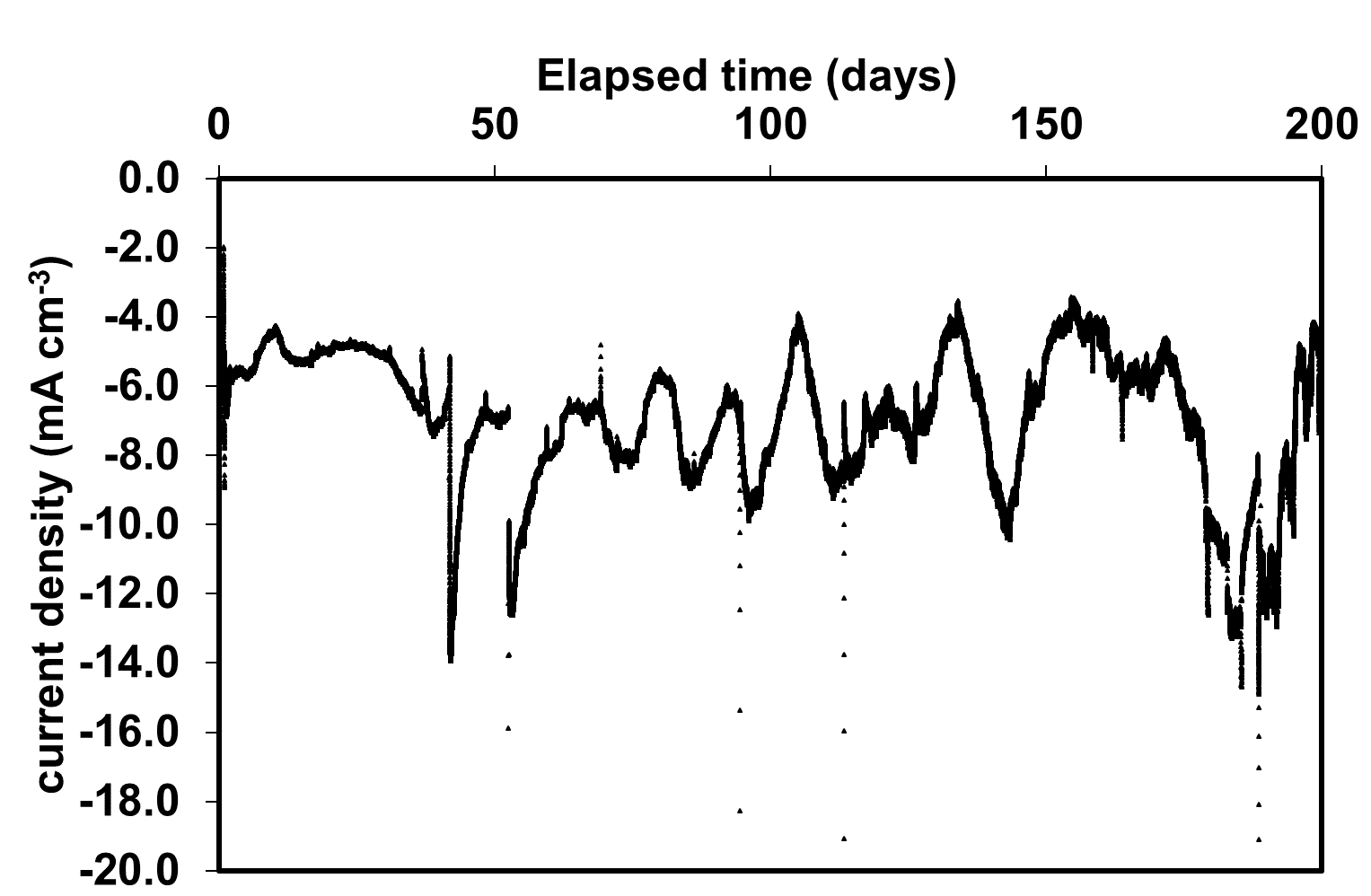


Figure S 9. Volume-specific current density of R1.


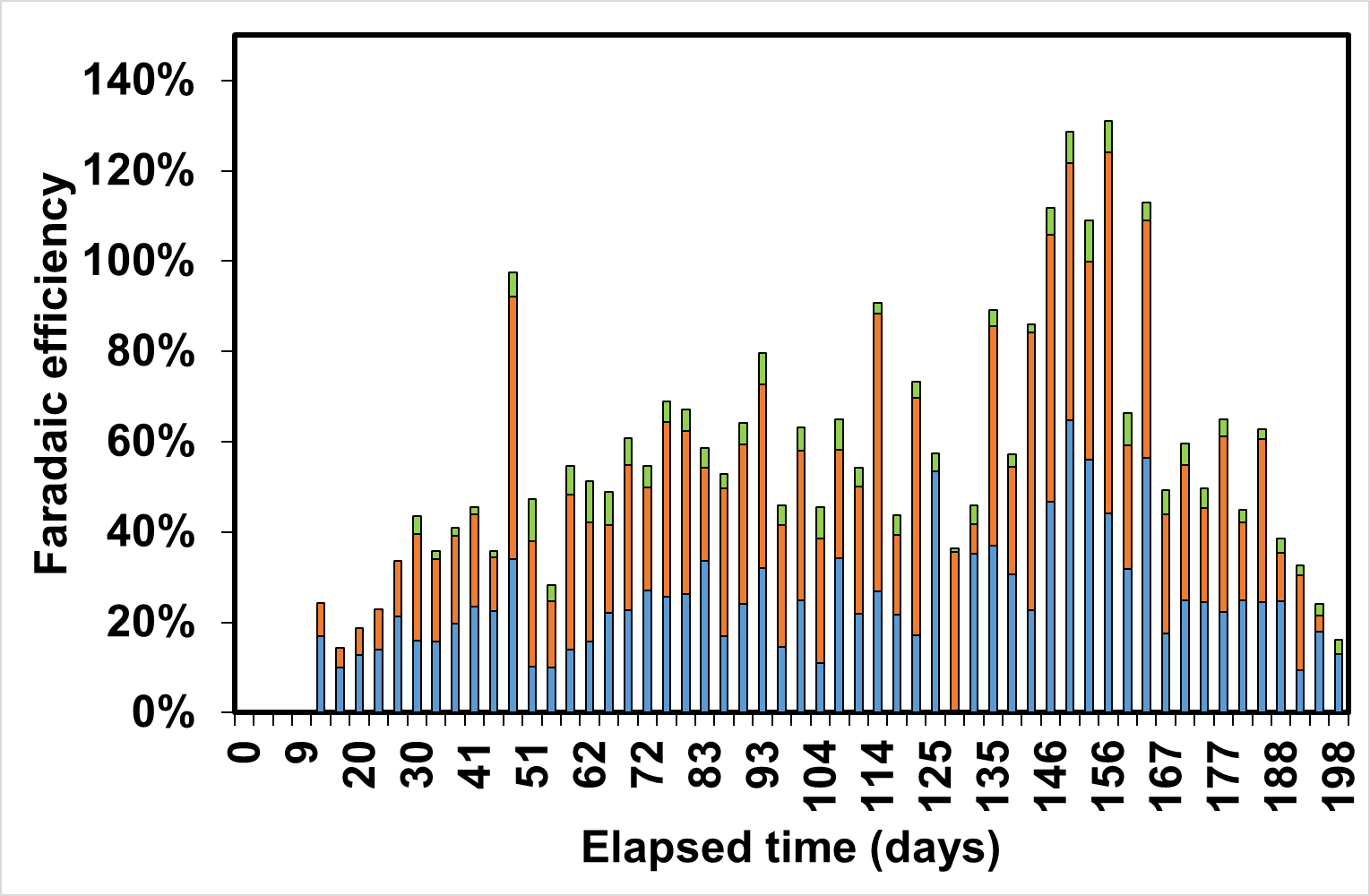


Figure S 10.. Faradaic efficiency R1. Colour code: blue is acetate, oranges is butyrate, and green is hexanoate

## Performance of R2


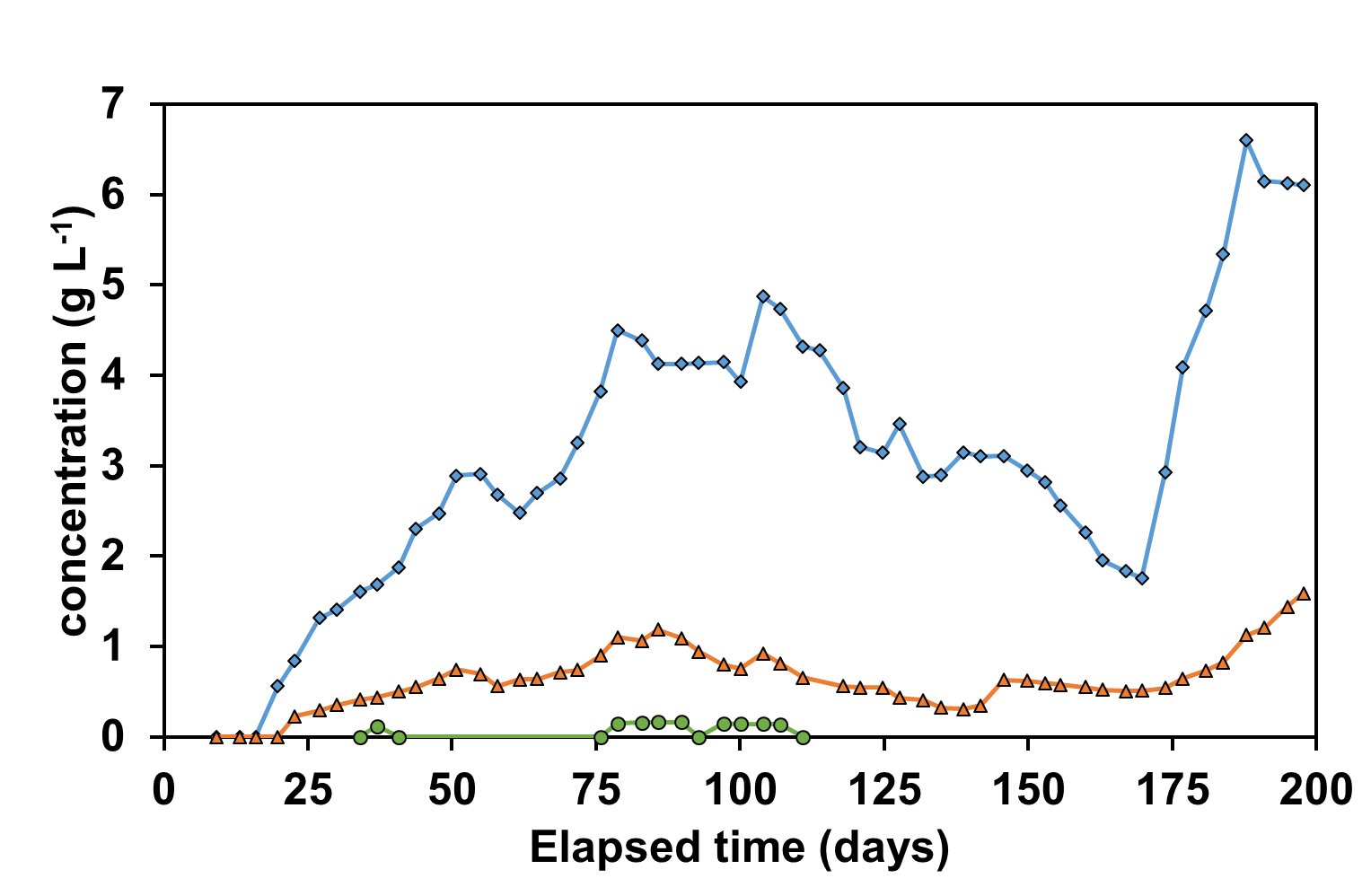


Figure S 11: concentration of organics R2. Colour code: blue diamonds is acetate, orange squares is butyrate, and green circles is hexanoate


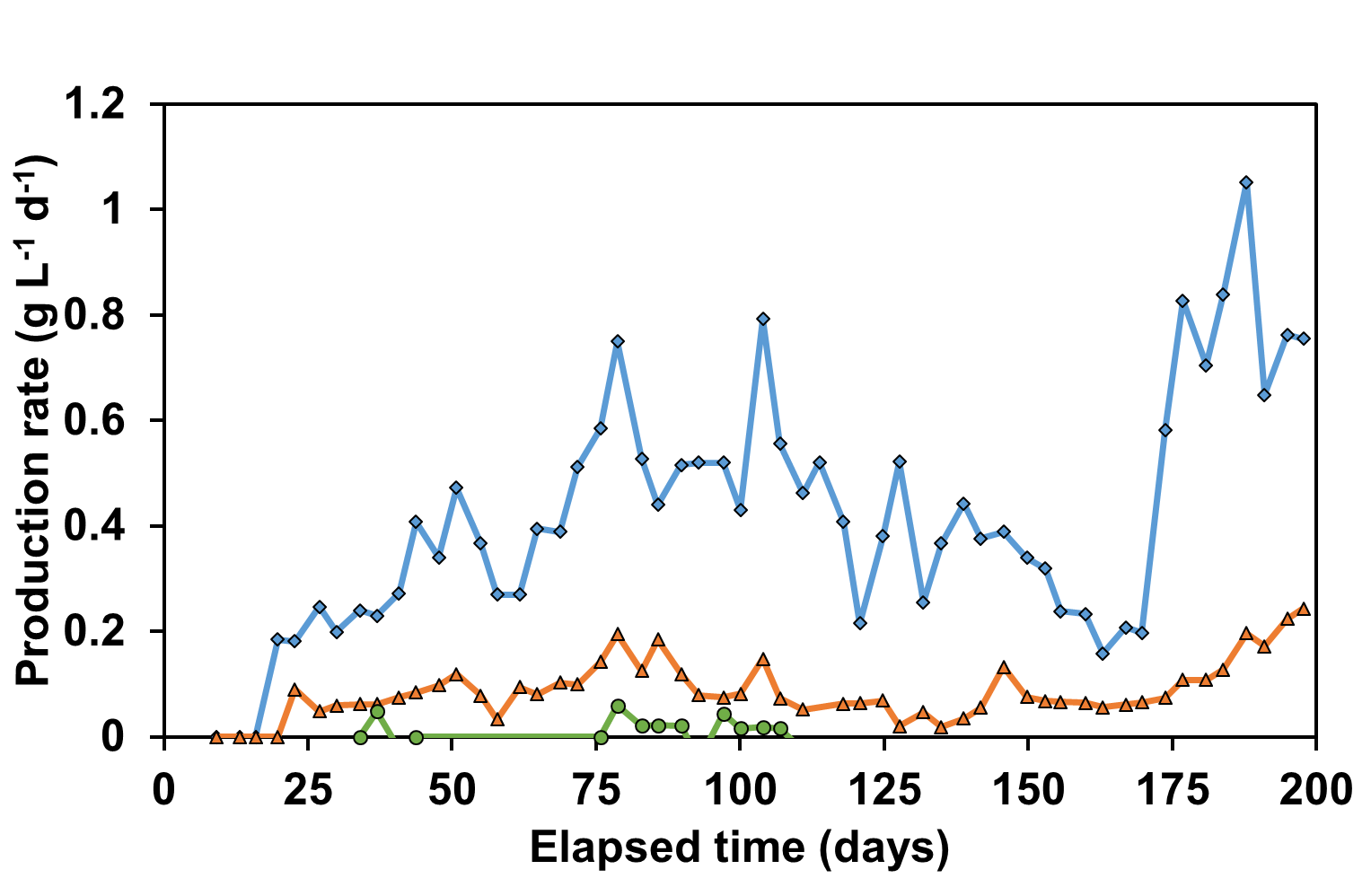


Figure S 12. Volume-specific production rate normalized to total catholyte volume in R2. Colour code: blue diamonds is acetate, orange squares is butyrate, and green circles is hexanoate


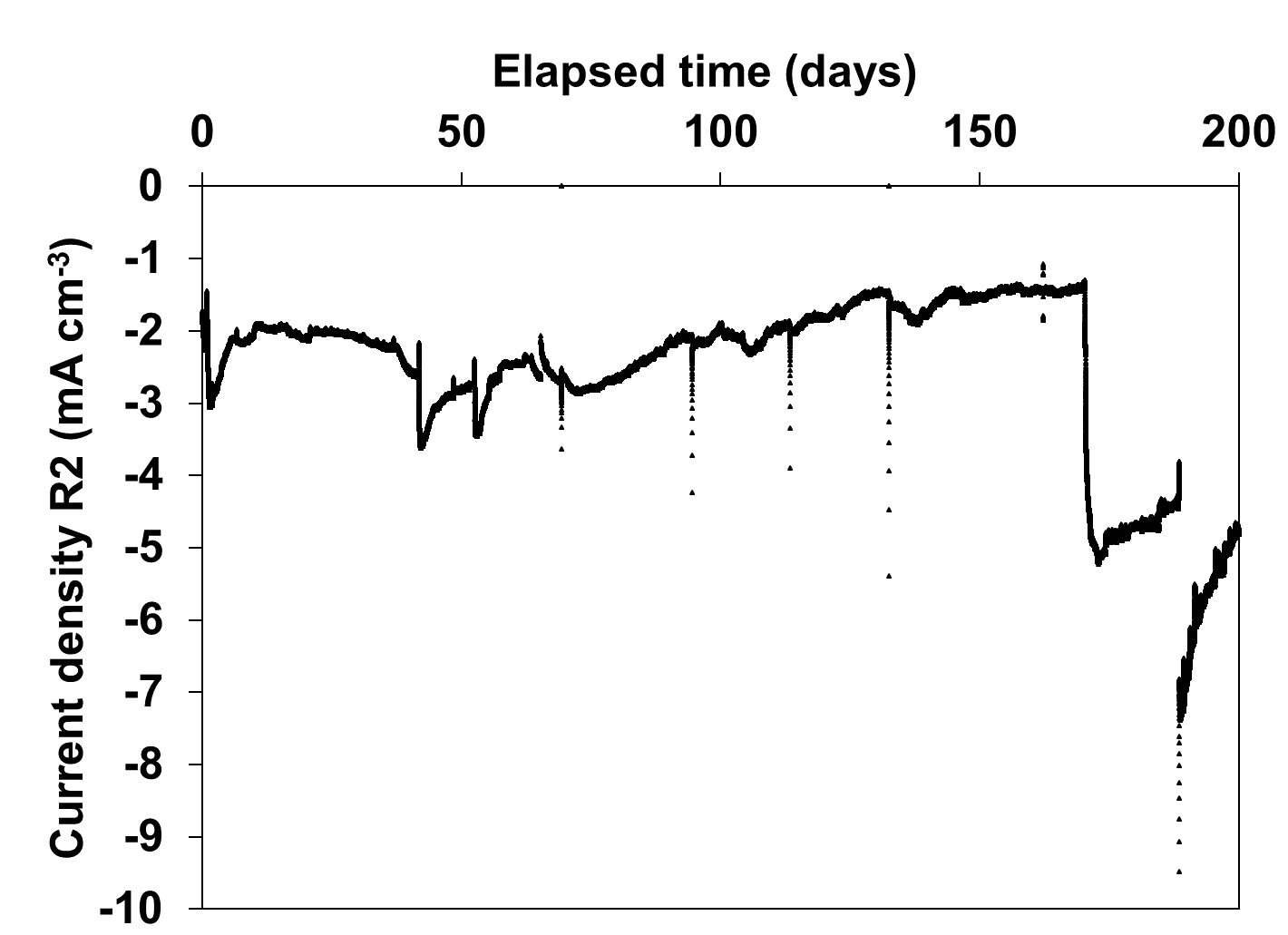


Figure S 13: Volume-specific current density of R2.


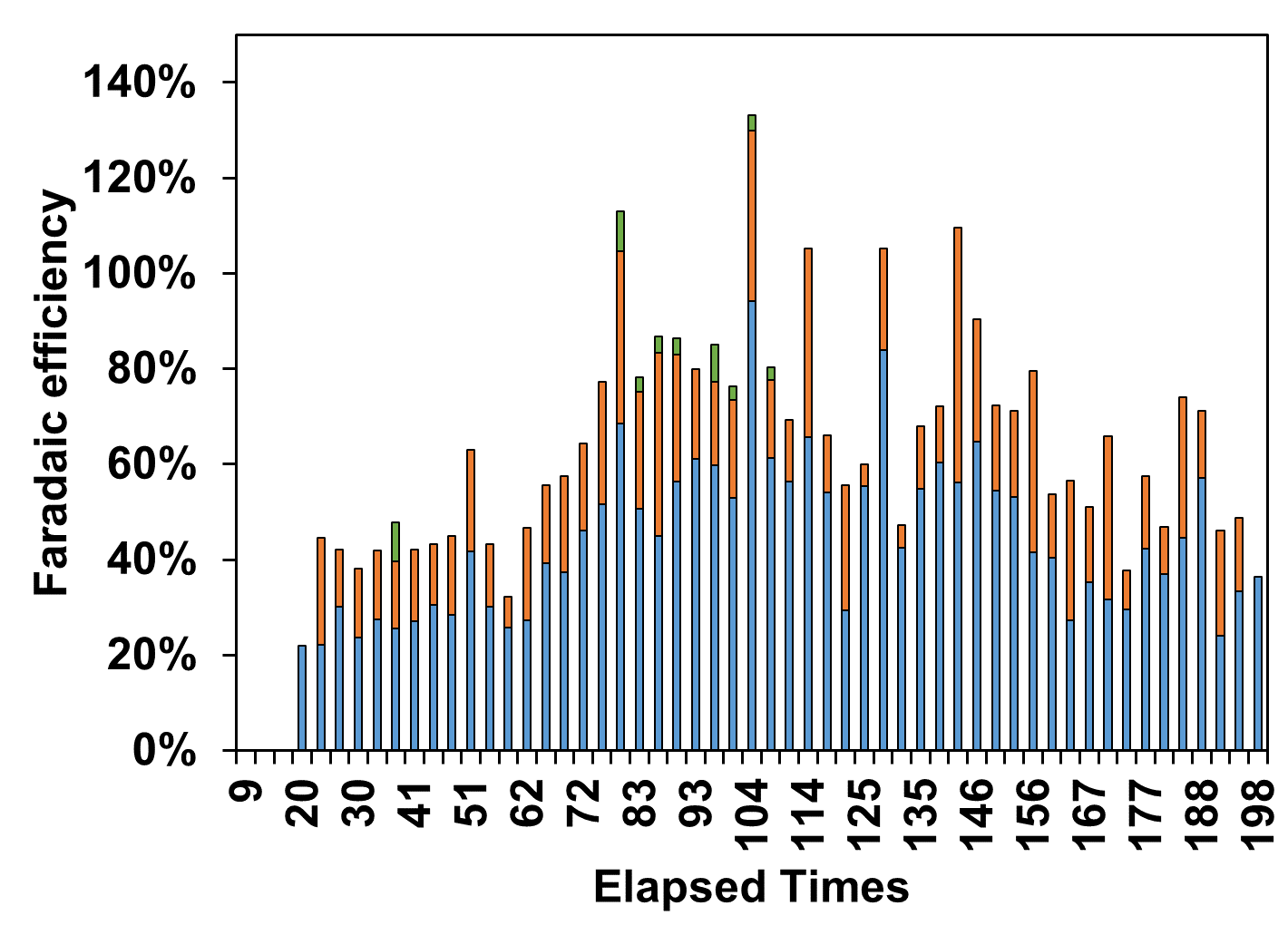


Figure S 14. Faradaic efficiency R2. Colour code: blue is acetate, orange is butyrate, and green is hexanoate

## Reactor performance of R3

##
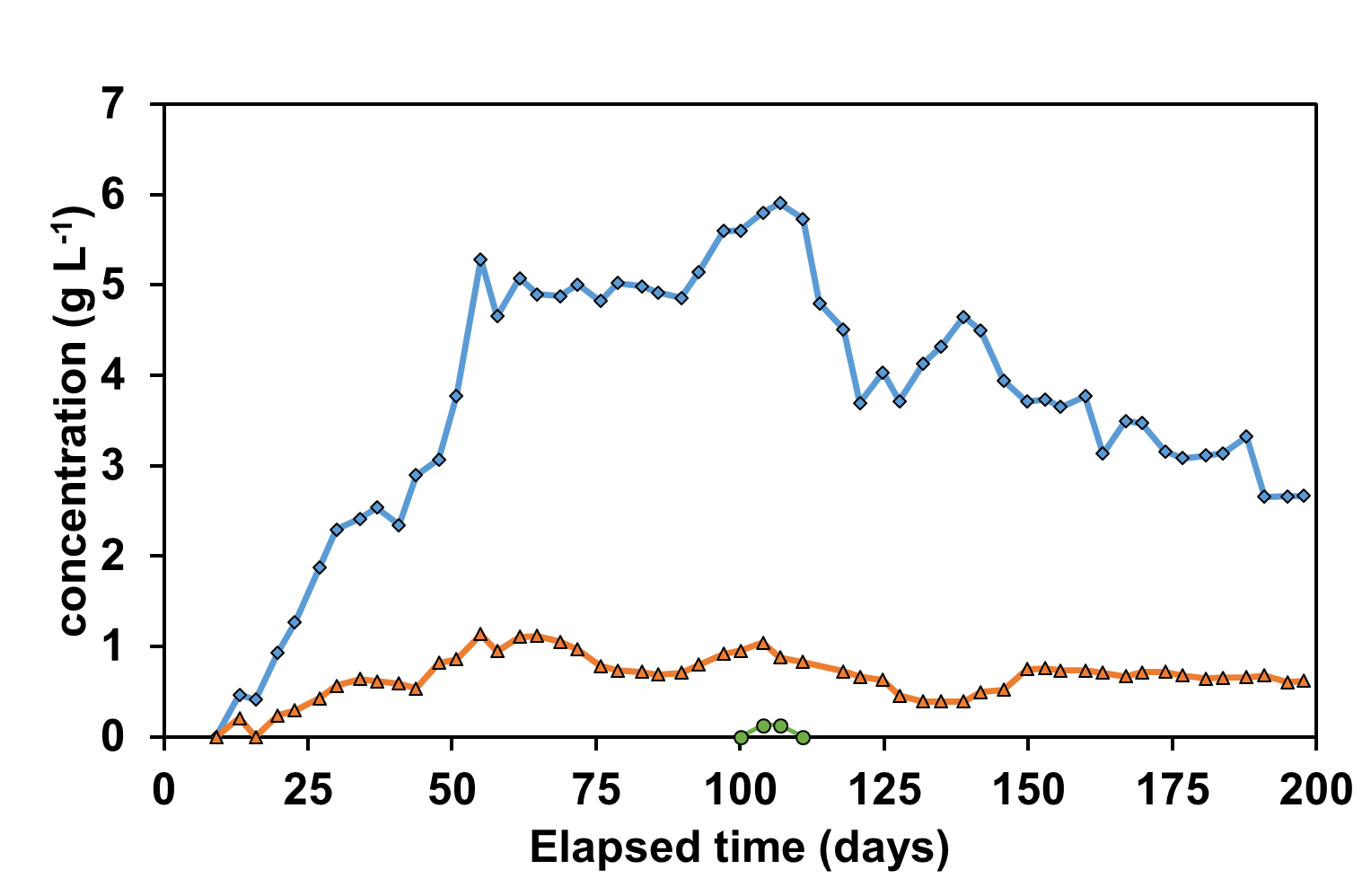


Figure S 15. Concentration of organics R3. Colour code: blue is acetate, orange is butyrate, and green is hexanoate


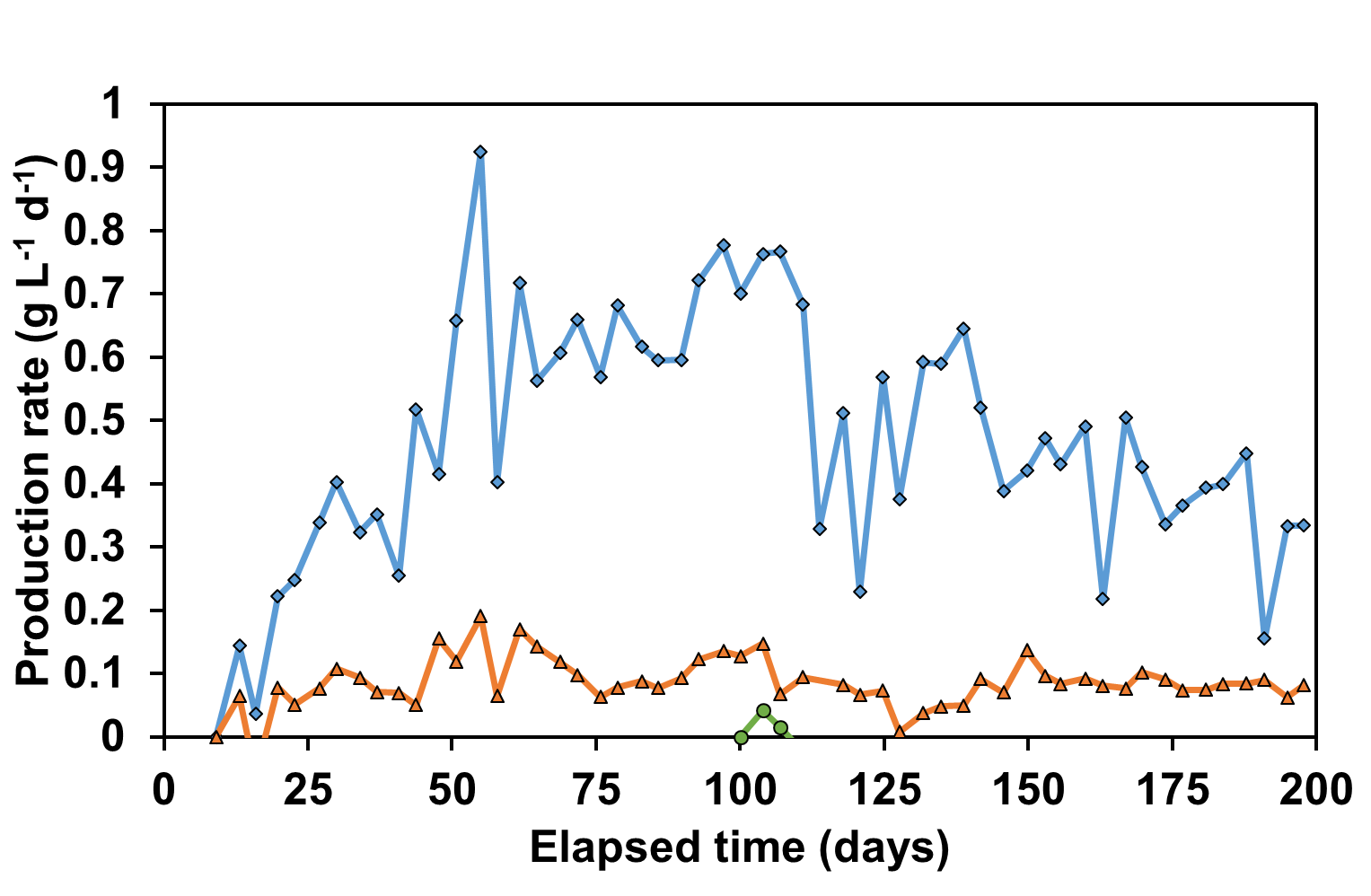


Figure S 16. Volume-specific production rate normalized to total catholyte volume in R3. Colour code: blue diamonds is acetate, orange squares is butyrate, and green circles is hexanoate


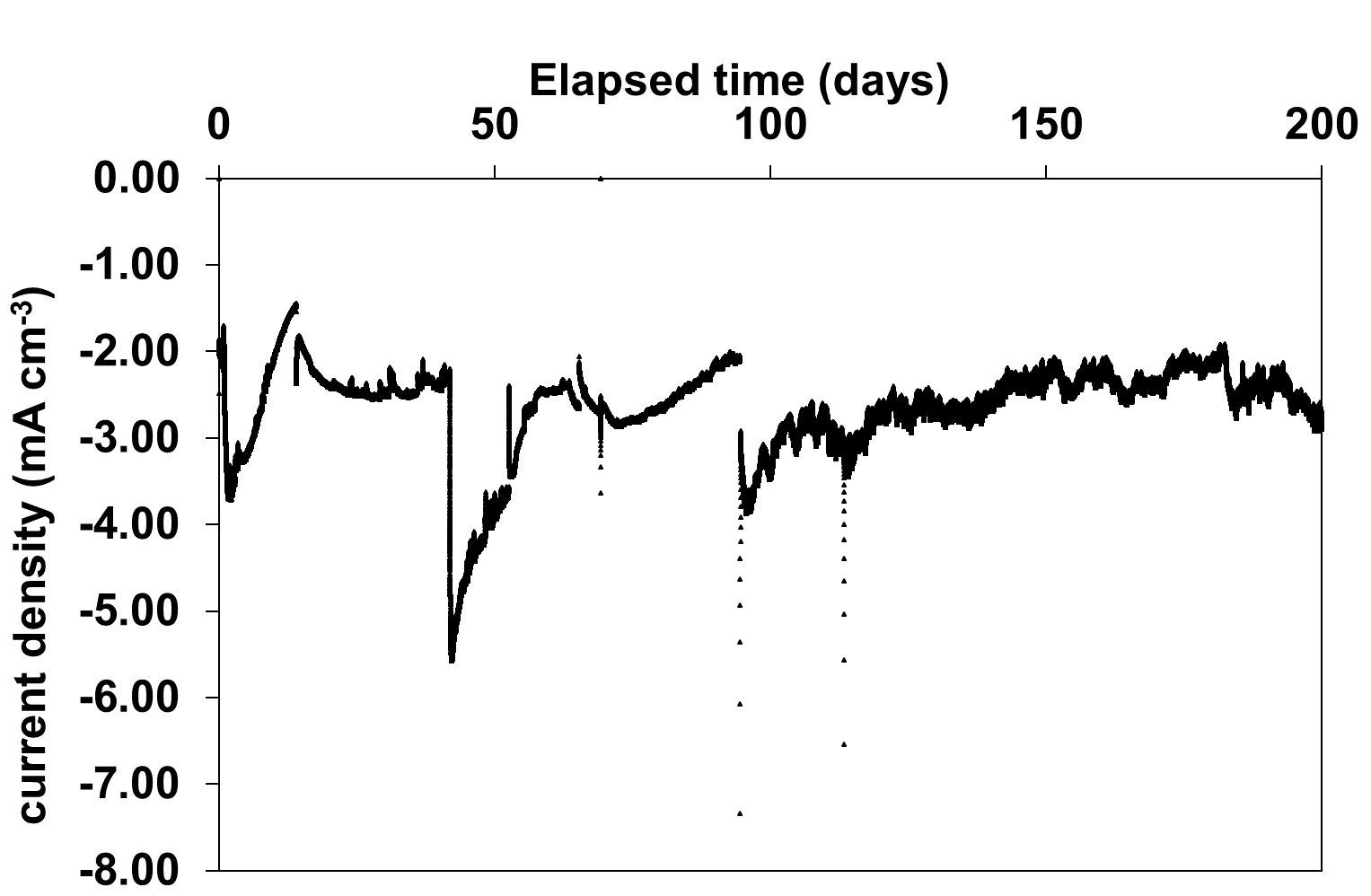


Figure S 17. Volume-specific current density R3


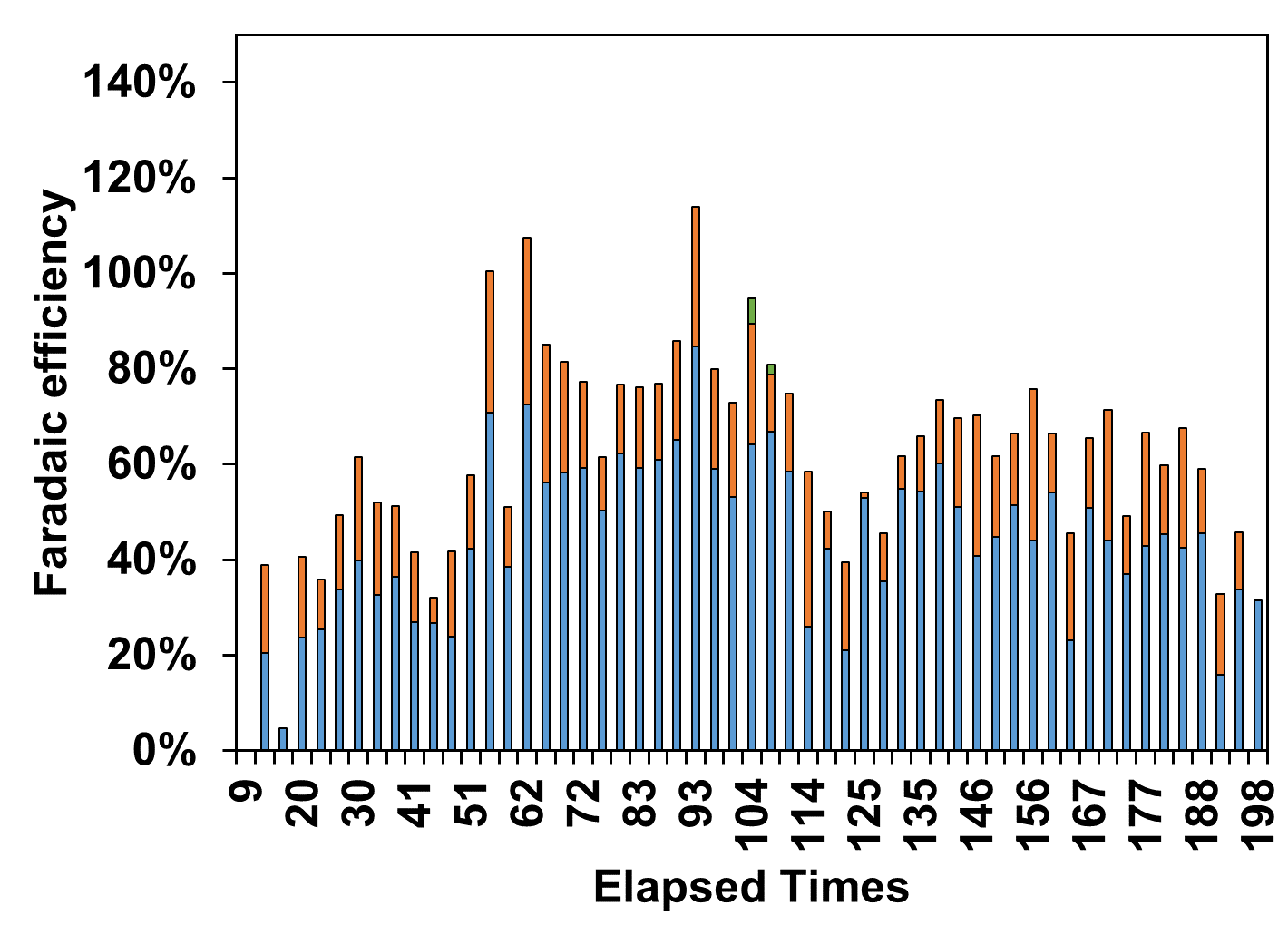


Figure S 18: Faradaic efficiency R3. Colour code: blue is acetate, orange is butyrate, and green is hexanoate

## Reactor performance of R4


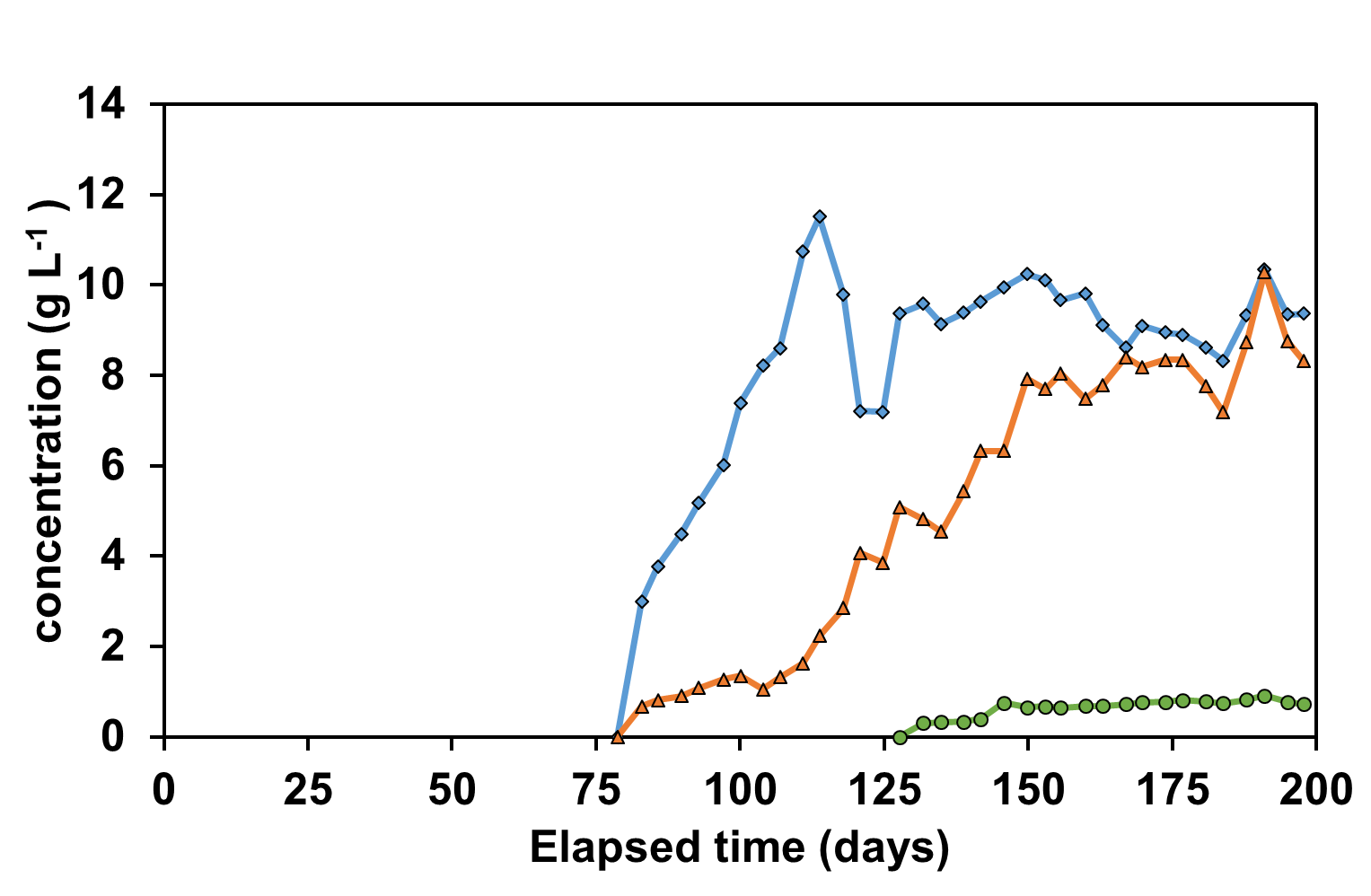


Figure S 19. Concentration of organics R4. Colour code: blue diamonds is acetate, orange squares is butyrate, and green circles is hexanoate


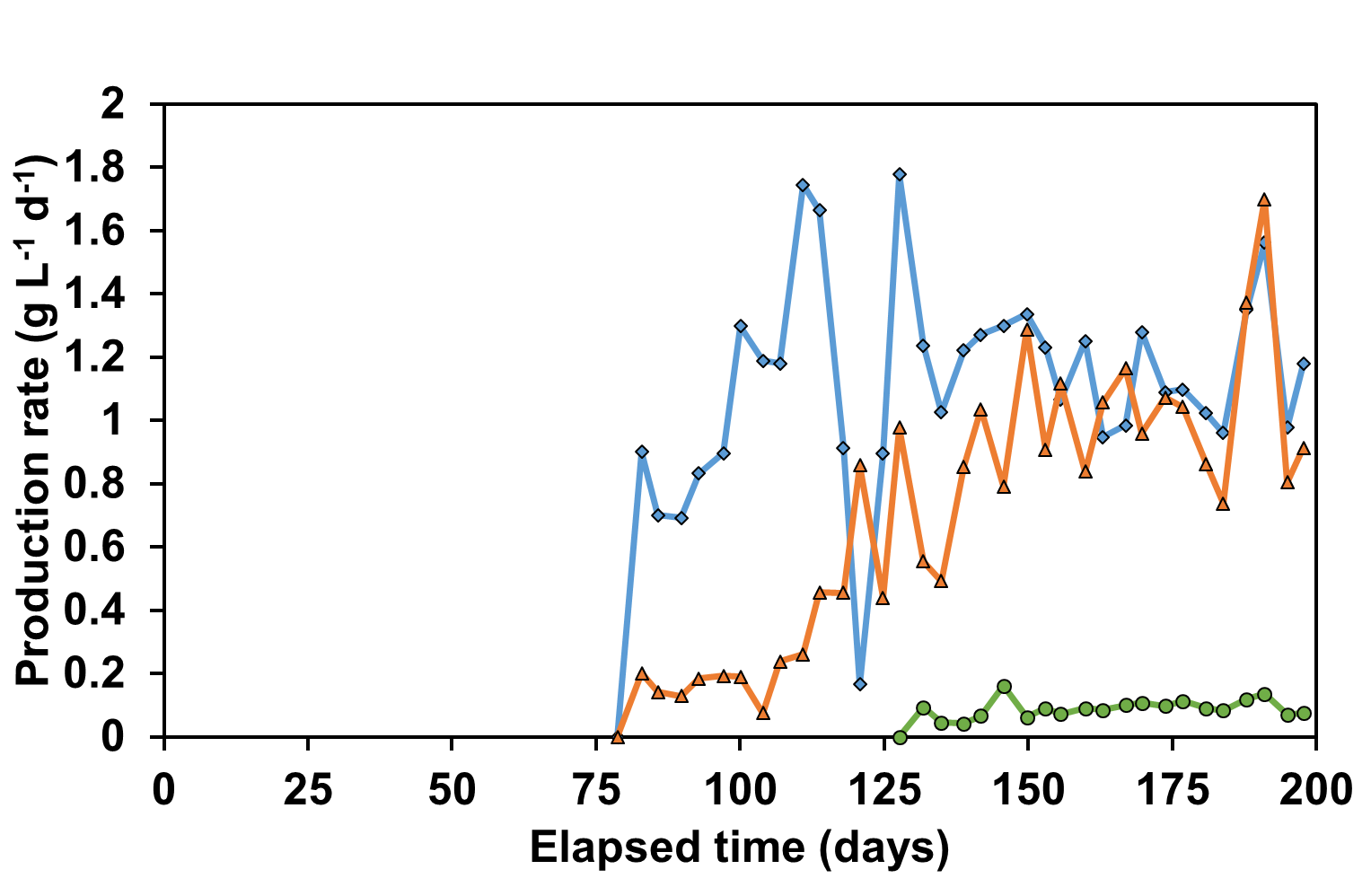


Figure S 20. Volume-specific production rate normalized to total catholyte volume in R4. Colour code: blue diamonds is acetate, orange squares is butyrate, and green circles is hexanoate


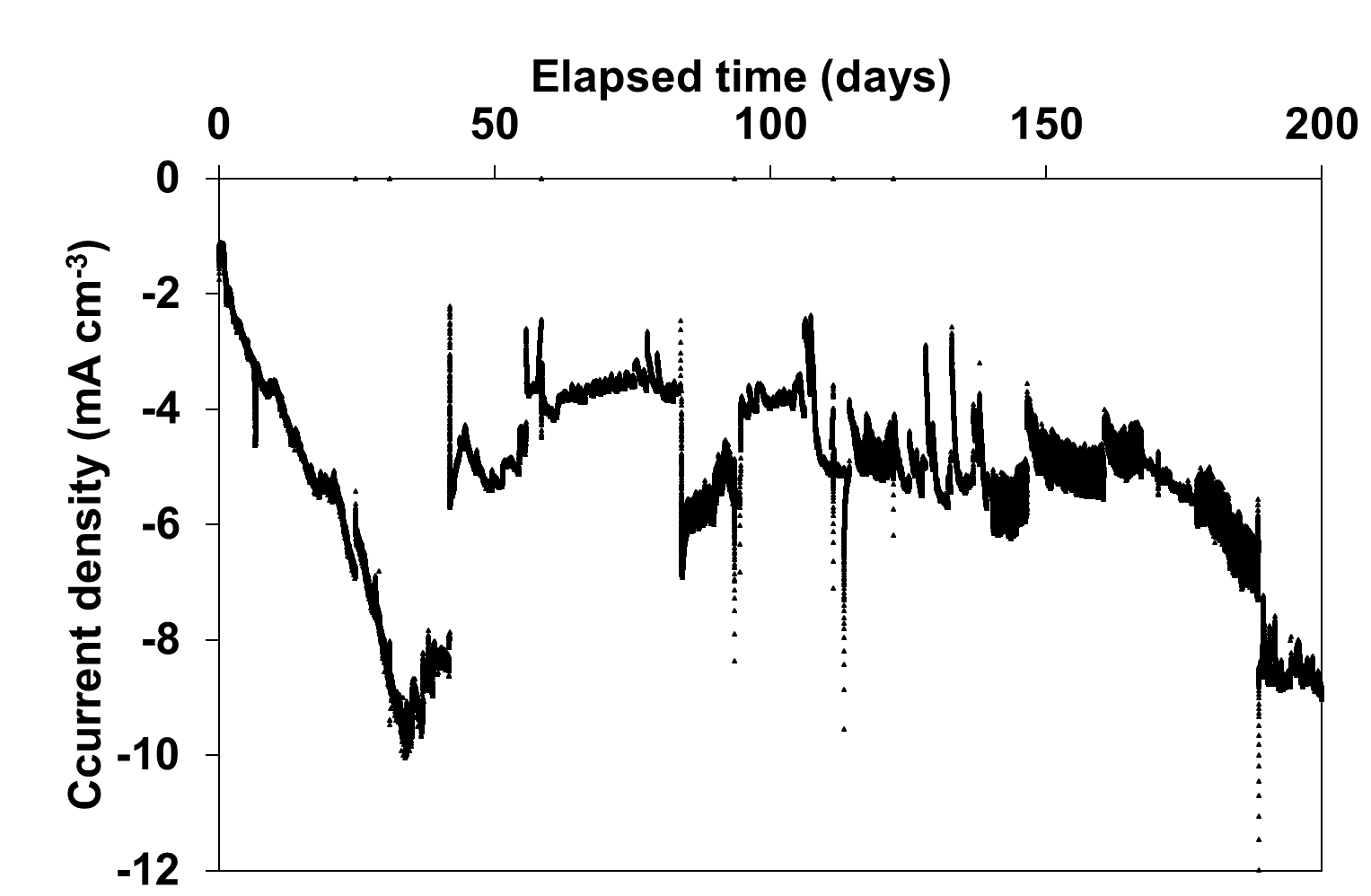


Figure S 21. Volume-specific current density R4


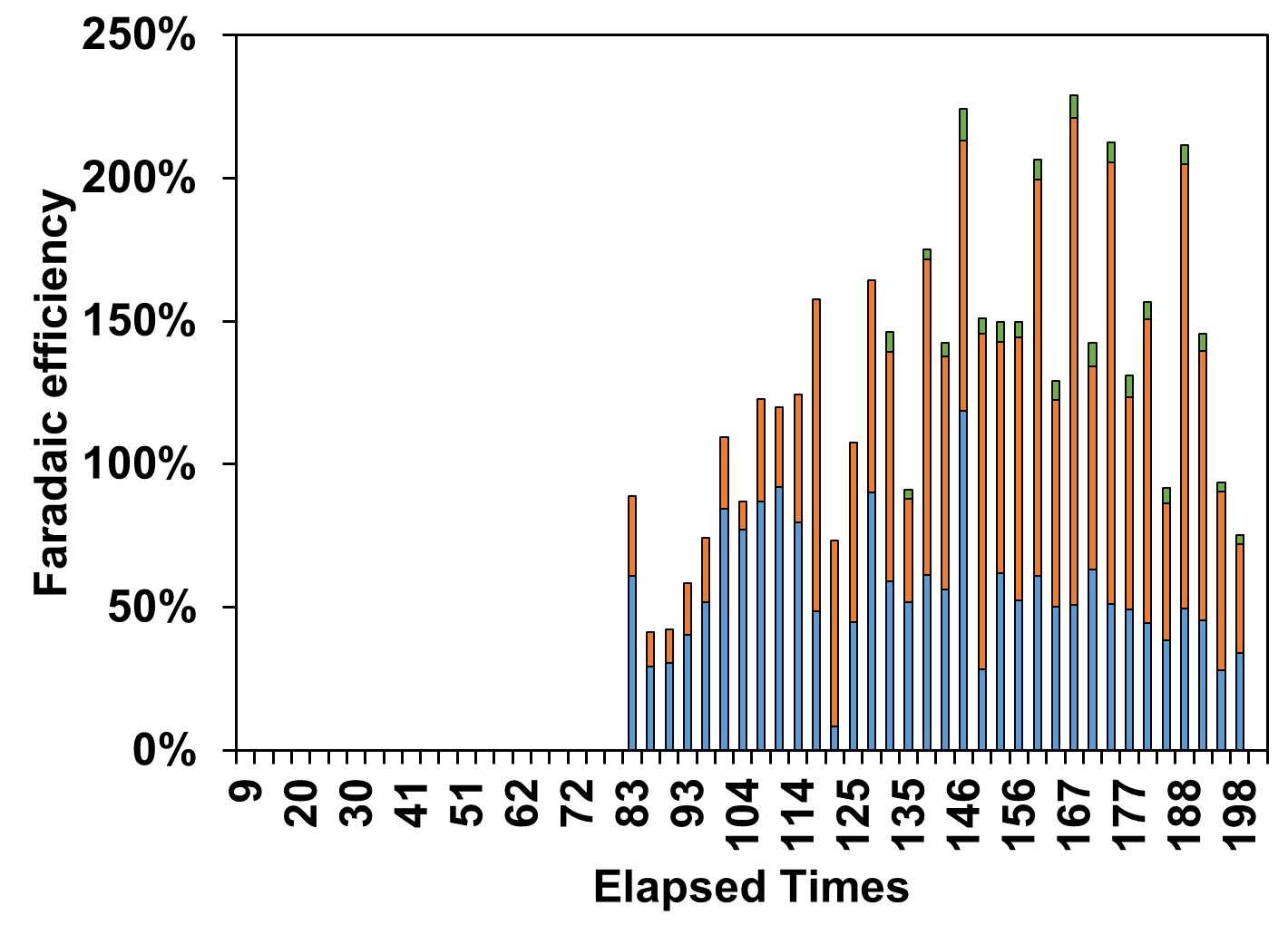


Figure S 22. Faradaic efficiency R4. Colour code: blue is acetate, orange is butyrate, and green is hexanoate ^1^

^1^The authors recognize the high improbability of the faradaic efficiencies of >100% obtained from day 100 onwards in R4. However, no flaws were discovered in the calculations, GC analysis, potentiostat channel or reference electrode. No additional electron donor was introduced to the reactor. One could speculate electron retention in the form of biomass and/or an unknown product between day 0 and day 83 which is then metabolized to organics between day 83-198. The faradaic efficiency over the whole experiment in R4 is 57%. 2-BES cannot be the responsible carbon and electron donor. Rago et al (2015) showed degradation of 2-BES in their aerobic microbial fuel cell system, but no degradation occurred in their anaerobic microbial electrolysis system. Moreover, hypothetical full consumption of 2-BES (4.5 g/L at 15 mL/d inflow) would result in an additional current of 3.2 mA, which is insufficient to explain the coulombic efficiency excess.”

# Concentration, production rates and current densities with conventional normalizations

Table S 3: Concentration and production rate normalizations. Selected time periods for R1: days 71-101, for R2: days 75-118, for R3 days 54-92, and for R4 days 141-198. The total surface area is calculated using carbon felt characteristics provided by the supplier: a specific area of 0.7 m^2^ g^-1^ and a density of 0.09 g cm^-3^

|  |  | Concentration | | Production rates | | | | | |
| --- | --- | --- | --- | --- | --- | --- | --- | --- | --- |
|  |  | **g L^-1^** | **mmol L^-1^** | **Catholyte volume**  **(g L^-1^ d^-1^)** | **Catholyte volume**  **(mmol L^-1^ d^-1^ )** | **Electrode volume**  **(g L_cathode_^-1^ d^-1^)** | **Projected surface area**  **(g m^-2^_PSA_ d^-1^)** | **Total surface area**  **(g m^-2^_TSA_ d^-1^)** | **Biomass specific**  **(mol mol_x_^-1^ d^-1^ )** |
| R1 | C_2_ | 5.71±0.32 | 96.7±5.4 | 0.74±0.13 | 12.6±2.2 | 12.1±2.1 | 121±21 | 0.193±0.033 | 0.046±0.010 |
|  | C_4_ | 3.62±0.36 | 41.6±4.2 | 0.48±0.11 | 5.47±1.30 | 7.78±1.85 | 77.7±18.5 | 0.123±0.029 | 0.020±0.005 |
|  | C_6_ | 0.078±0.008 | 6.81±0.74 | 0.094±0.017 | 0.81±0.15 | 1.52±0.28 | 15.2±2.8 | 0.024±0.004 | 0.0029±0.0005 |
| R2 | C_2_ | 4.25±0.32 | 72.0±5.4 | 0.54±0.11 | 9.2±1.9 | 8.8±1.9 | 88±19 | 0.140±0.030 | 0.031±0.008 |
|  | C_4_ | 0.87±0.21 | 10.0±2.4 | 0.11±0.05 | 1.24±0.55 | 1.76±0.78 | 17.6±7.8 | 0.028±0.012 | 0.004±0.002 |
|  | C_6_ | 0.09±0.08 | 0.80±0.66 | 0.011±0.027 | 0.10±0.23 | 0.18±0.44 | 1.8±4.4 | 0.003±0.007 | 0.0004±0.0008 |
| R3 | C_2_ | 4.96±0.16 | 84.1±2.8 | 0.64±0.12 | 10.8±2.1 | 10.4±2.0 | 104±20 | 0.165±0.032 | 0.048±0.018 |
|  | C_4_ | 0.90±0.18 | 10.3±2.0 | 0.11±0.04 | 1.25±0.47 | 1.78±0.67 | 17.8±6.8 | 0.028±0.011 | 0.006±0.003 |
|  | C_6_ | n.a. | n.a. | n.a. | n.a. | n.a. | n.a. | n.a. | n.a. |
| R4 | C_2_ | 9.37±0.60 | 159±10 | 1.17±0.17 | 19.8±2.9 | 19.1±2.8 | 191±28 | 0.303±0.045 | 0.048±0.008 |
|  | C_4_ | 7.99±0.92 | 91.9±10.6 | 1.04±0.24 | 11.9±2.8 | 17.0±4.0 | 170±40 | 0.269±0.063 | 0.029±0.008 |
|  | C_6_ | 0.72±0.11 | 6.29±0.95 | 0.095±0.026 | 0.83±0.23 | 1.55±0.43 | 15.5±4.3 | 0.025±0.007 | 0.0020±0.0006 |

Table S 4: Current density normalizations and cell voltages. Selected time periods for R1: days 71-101, for R2: days 75-118, for R3 days 54-92, and for R4 days 141-198. The total surface area is calculated using carbon felt characteristics provided by the supplier: a specific area of 0.7 m^2^ g^-1^ and a density of 0.09 g cm^-3^

|  | Current Density normalizations | | | |  |
| --- | --- | --- | --- | --- | --- |
|  | Projected surface area  (A m^-2^_PSA_) | Total surface area  (A m^-2^_TSA_) | Cathode volume  (kA m ^-3^) | Biomass-specific current  (A mol_X_^-1^) | Cell voltage  (V) |
| R1 | -75.17±10.50 | -0.12±0.02 | -7.52±1.05 | 3.08±0.69 | -4.03±0.17 |
| R2 | -22.19±2.64 | -0.035±0.004 | -2.22±0.26 | 0.83±0.44 | -3.12±0.06 |
| R3 | -25.55±2.08 | -0.041±0.003 | -2.55±0.21 | 1.34±0.56 | -3.12±0.05 |
| R4 | -58.56±13.39 | -0.094±0.021 | -5.86±1.34 | 1.62±0.47 | -3.64±0.17 |

# Biofilm versus planktonic cells retained in the systems over time

Figure S 21-24 show the ratio of biofilm versus planktonic cells in the reactors until day 70, with the exception of R2 (Figure S 22) which shows the ratio until day 197. Reactor 2 showed an increase in the planktonic cells ratio with a peak of 3.6% on day 174, whereas the other reactors remained <1% in planktonic cells ratio until the end of the experiment. Day 70 was chosen for reactor R1, R3 and R4 for visualization purposes of the start-up phase.


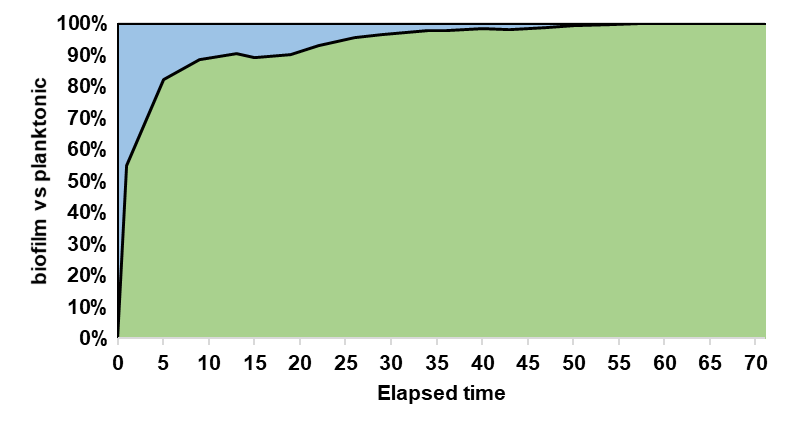


Figure S 23. R1 biofilm versus planktonic cells until day 70. The planktonic biomass is shown in blue and biofilm-based biomass is shown in green.


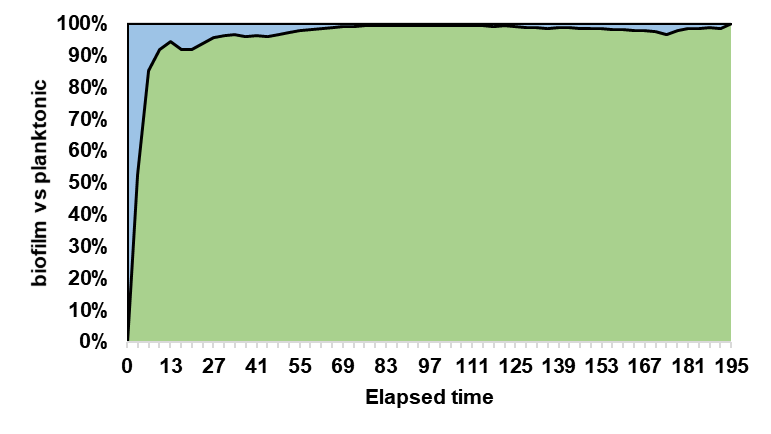


Figure S 24 R2 biofilm versus planktonic cells until day 197. The planktonic biomass is shown in blue and biofilm-based biomass is shown in green.


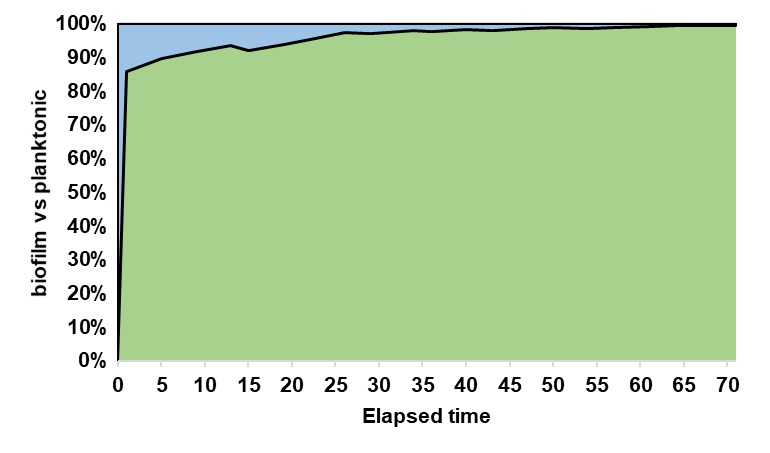


Figure S 25. R3 biofilm versus planktonic cells. The planktonic biomass is shown in blue and biofilm-based biomass is shown in green.


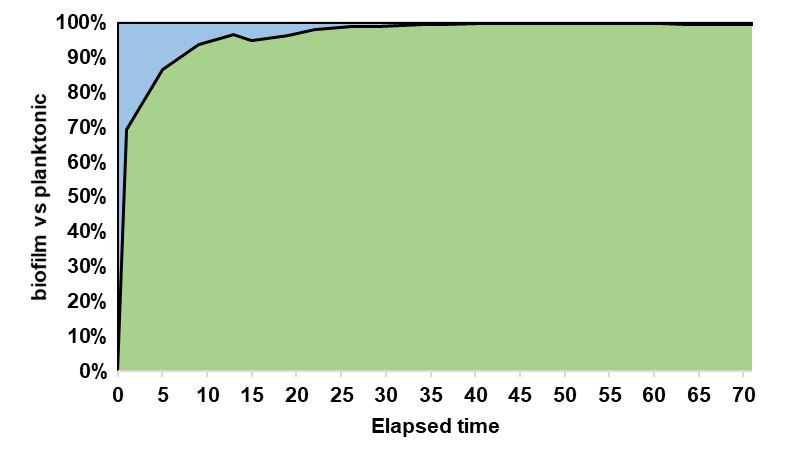


Figure S 26. R4 biofilm versus planktonic cells. The planktonic biomass is shown in blue and biofilm-based biomass is shown in green.

# Biofilm pictures


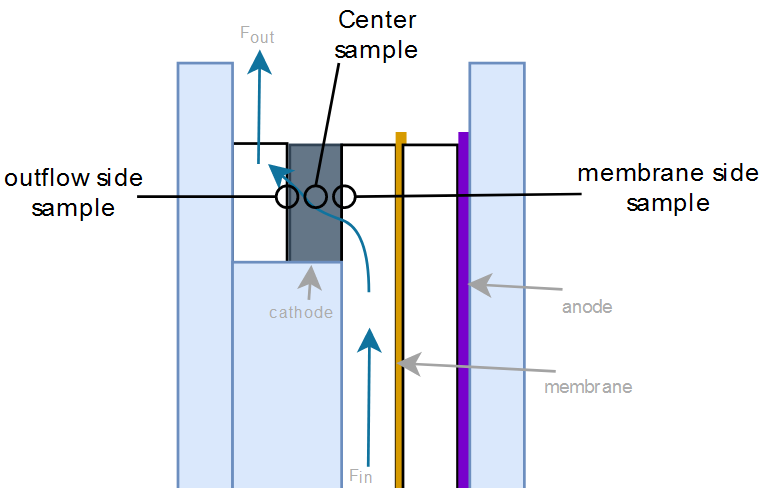


Figure S 27: schematic overview of biofilm sampling locations for imaging

## Biofilm images R1


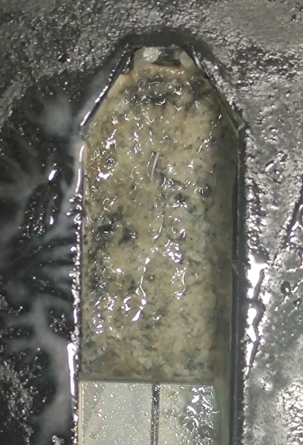

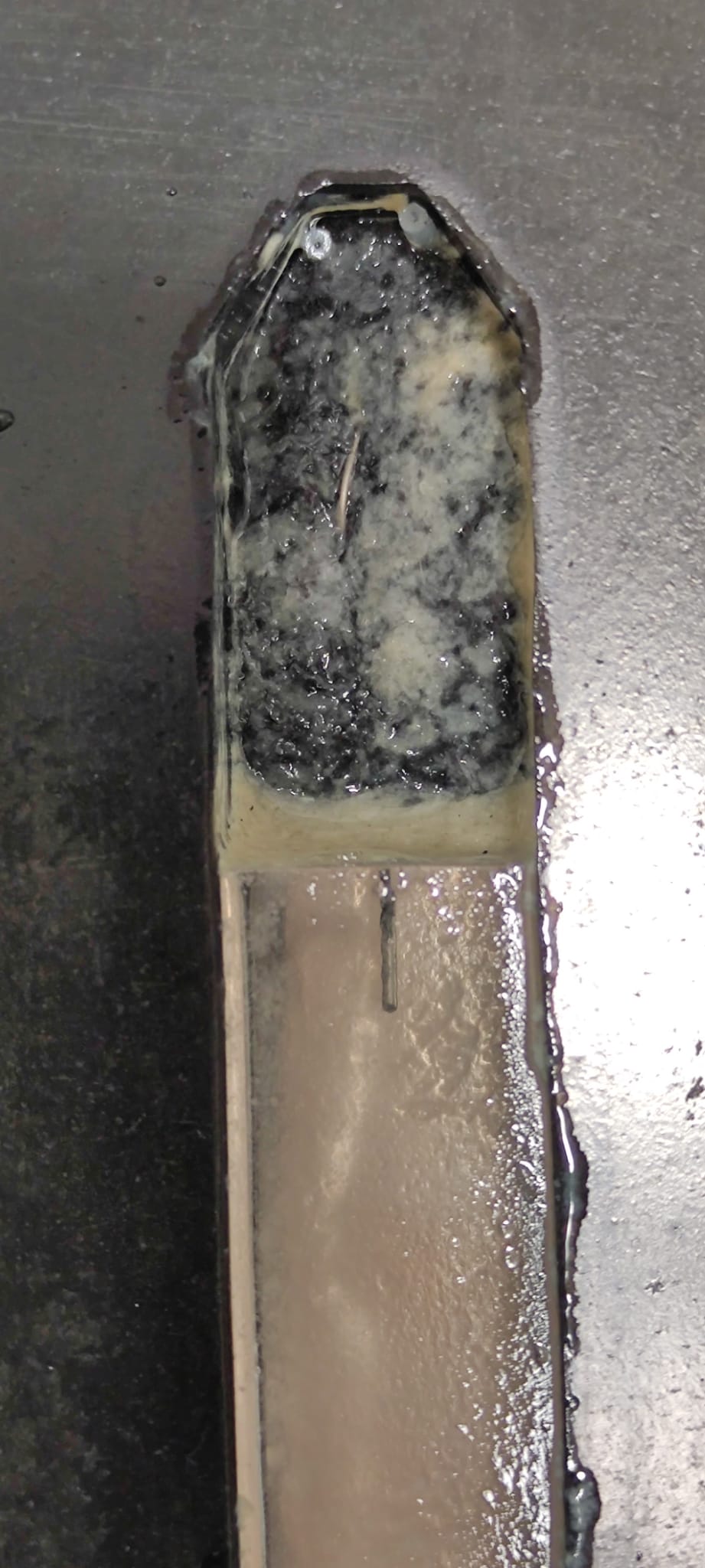


Figure S 28: Biofilm R1 membrane side Figure S 29 Biofilm R1 outflow side

## Biofilm images R2


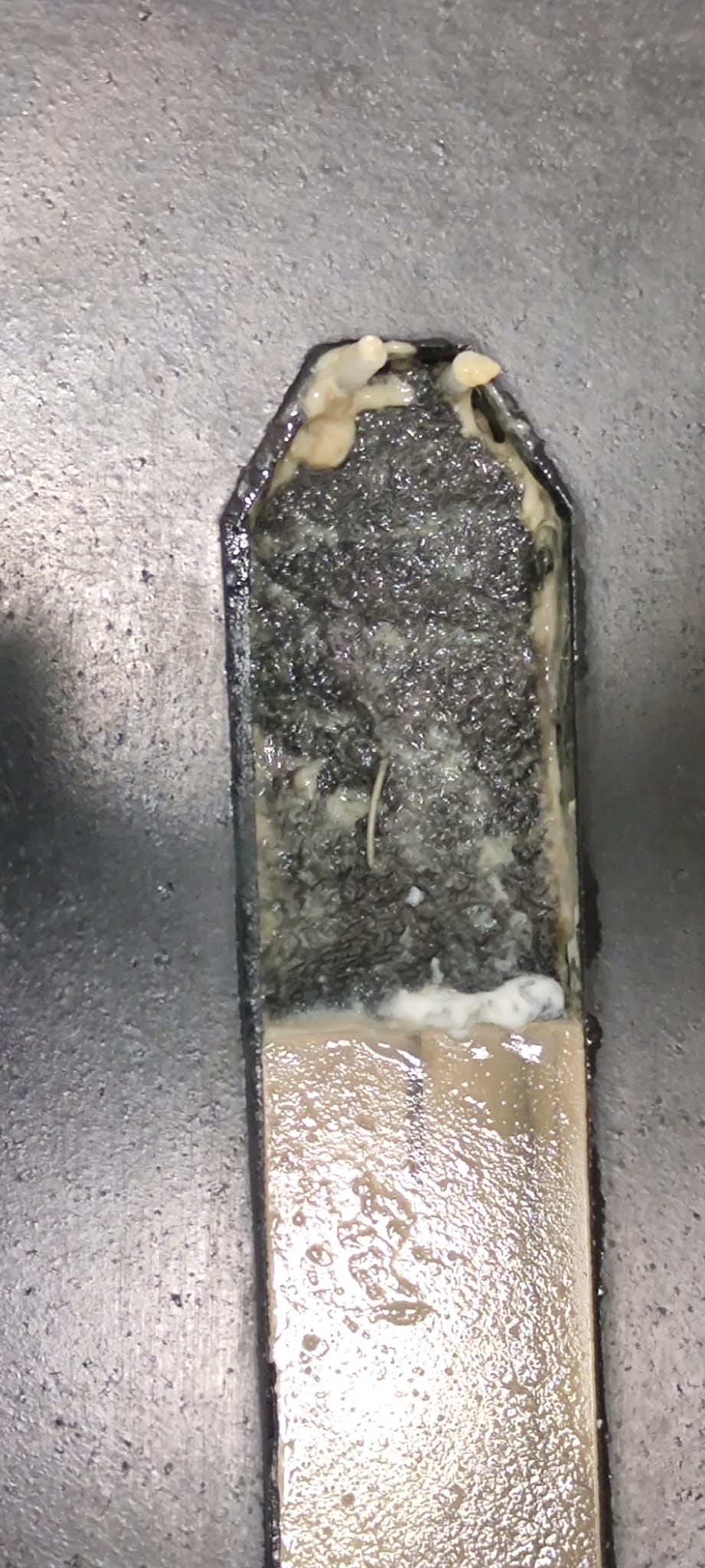

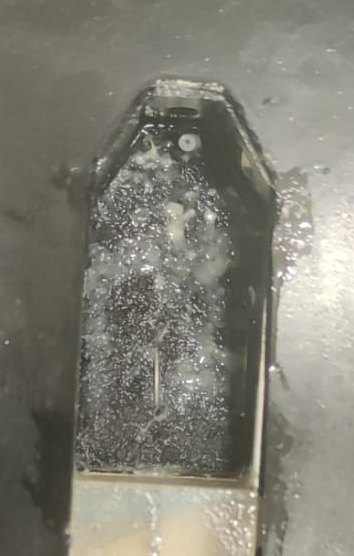


Figure S 30. Biofilm R2 membrane side Figure S 31. Biofilm R2 outflow side

## Biofilm images R3


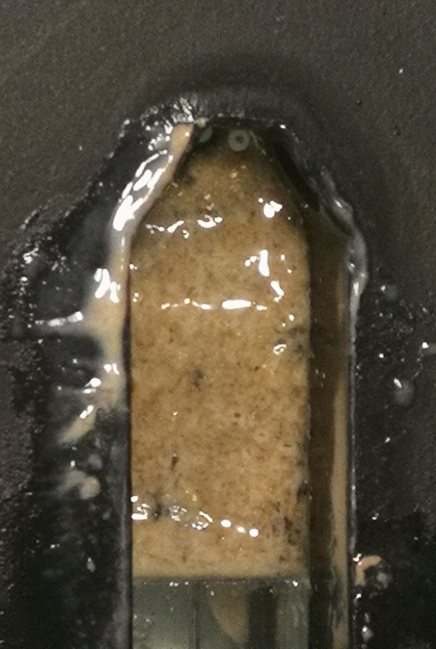


Figure S 32. Biofilm R3 membrane side. Picture of outflow side of R3 was not taken

## Biofilm images R4


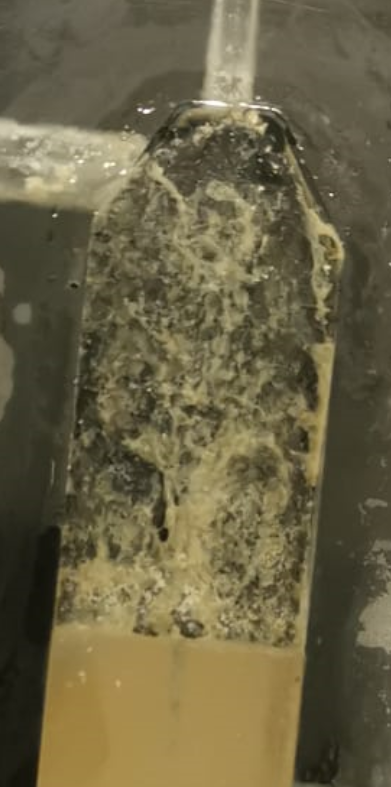

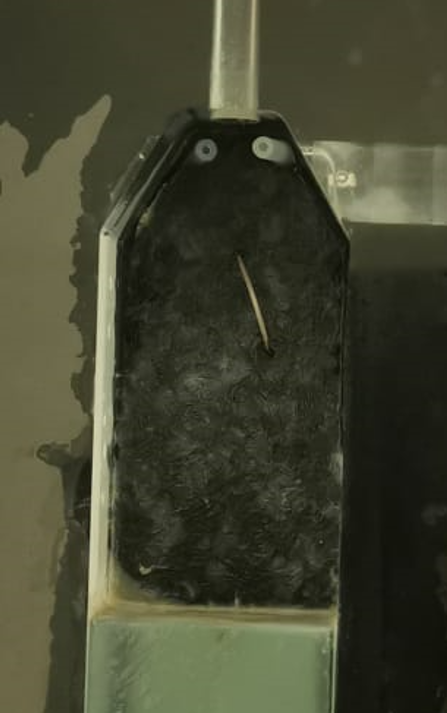


Figure S 33. Biofilm R4 membrane side. Figure S 34. Biofilm R4 outflow side.

# Live/dead staining images

## Live/dead R1


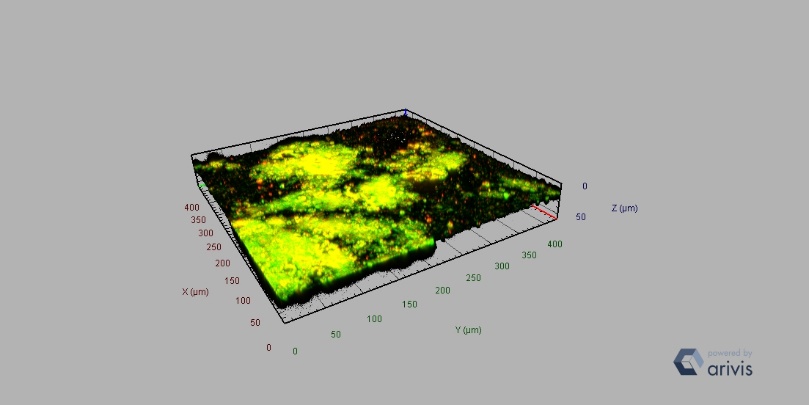


Figure S 35:R1 membrane side image. Z-stack: 42 slices (57.4 μm). 20x magnification


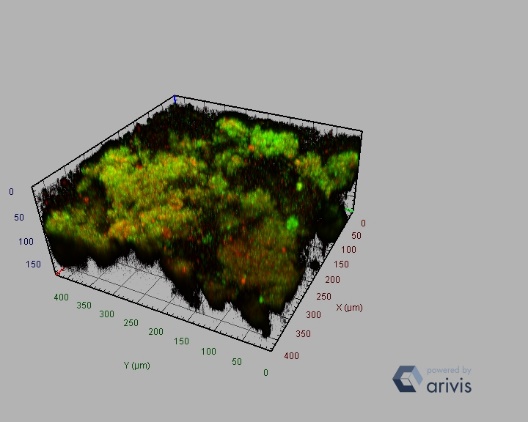


Figure S 36 R1 center sample image. Z-stack: 31 slices (180 μm). 20x magnification


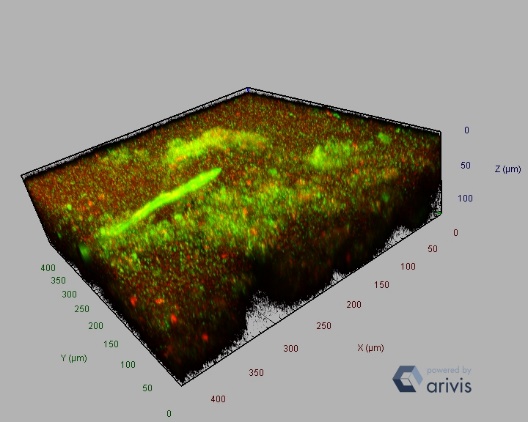


Figure S 37: R1 outflow side image. Z-stack: 41 slices (120 μm). 20x magnification

## Live/dead R2


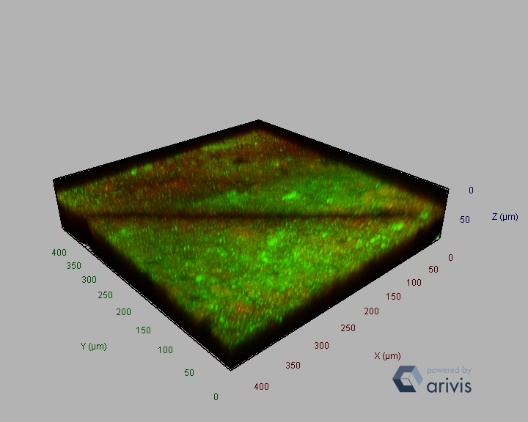


Figure S 38: R2 Membrane side image. Z-stack:29 slices (84 μm). 20x magnification


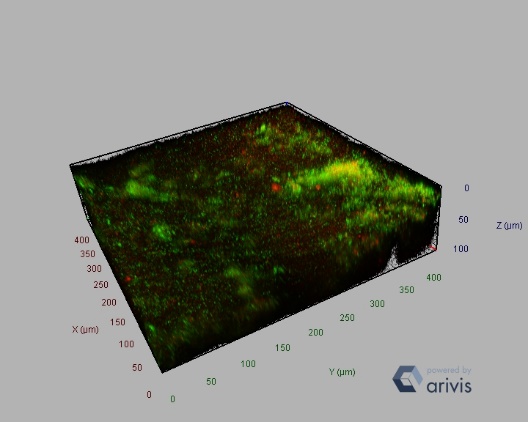


Figure S 39: R2 center sample image. Z-stack: 36 slices (105 μm). 20x magnification


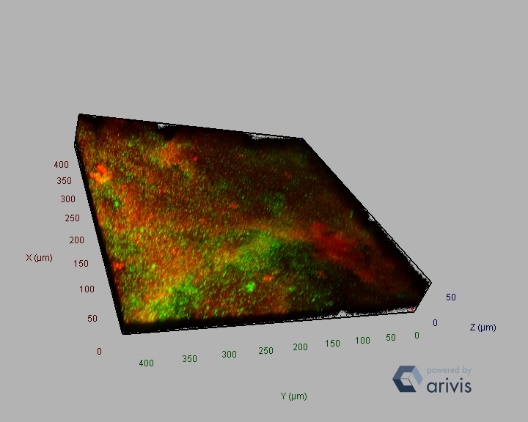


Figure S 40: R2 outflow side image. Z-stack: 19 slices (54 μm). 20x magnification

## Live/dead R3


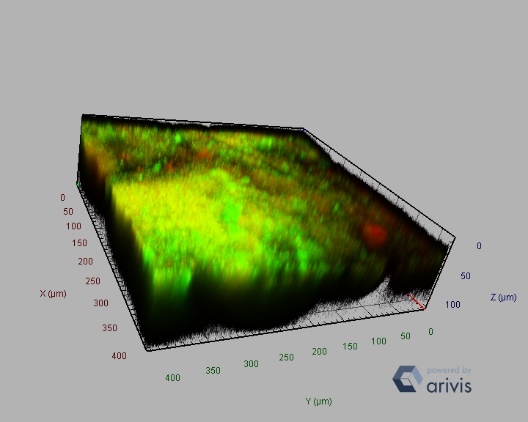


Figure S 41: R3 membrane side. Z-stack: 25 slices (120 μm). 20x magnification


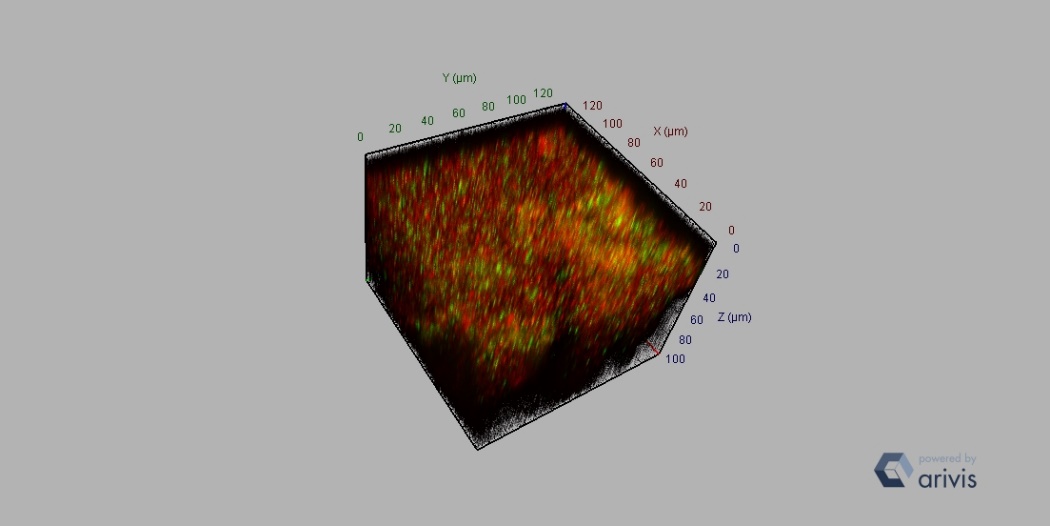


Figure S 42: R3 center sample. Z-stack: 20 slices (95 μm). 20x magnification


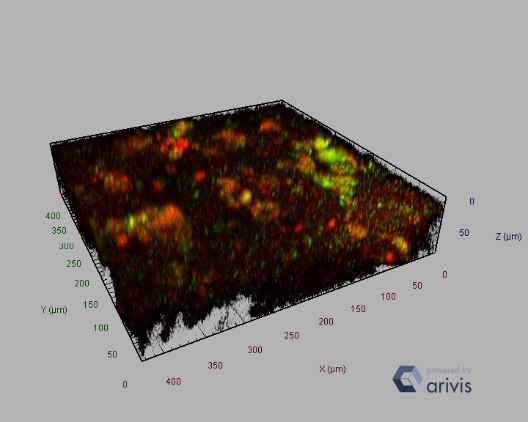


Figure S 43: R3 outflow side. Z-stack:18 slices (85 μm). 20x magnification

## Live/dead R4


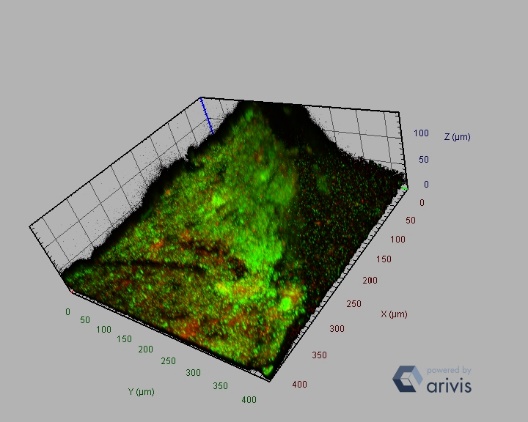


Figure S 44: R4 Membrane side . Z-stack: 49 slices 144 μm). 20x magnification


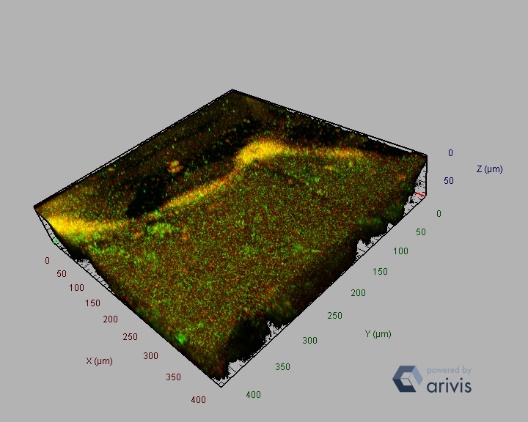


Figure S 45: R4 center sample. Z-stack: 51 slices (75 μm). 20x magnification


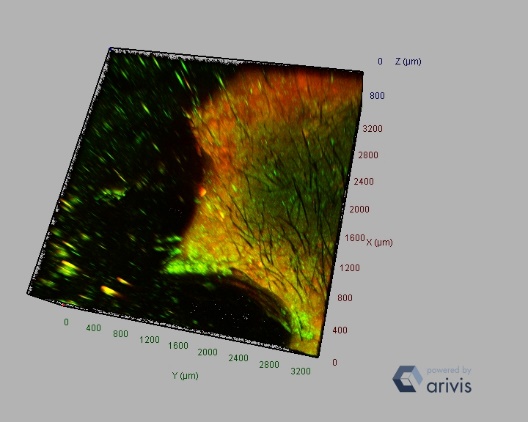


Figure S 46: R4 outflow side. Z-stack: 29 slices (840 μm). 2.5x magnification

# Supplementary material reference list

Rago, L., Guerrero, J., Baeza, J. A., & Guisasola, A. (2015). 2-Bromoethanesulfonate degradation in bioelectrochemical systems. Bioelectrochemistry, 105, 44-49.
